# Light Responsive Polymer Membranes: A Review

**DOI:** 10.3390/membranes2010134

**Published:** 2012-03-02

**Authors:** Fiore Pasquale Nicoletta, Daniela Cupelli, Patrizia Formoso, Giovanni De Filpo, Valentina Colella, Annarosa Gugliuzza

**Affiliations:** 1 Department of Pharmaceutical Sciences, Università della Calabria, I-87036 Rende (CS), Italy; Email: dcupelli@unical.it (D.C.); formoso@unical.it (P.F.); colella.va@libero.it (V.C.); 2 Department of Chemistry, Università della Calabria, I-87036 Rende (CS), Italy; Email: defilpo@unical.it; 3 Institute on Membrane Technology—National Council Research, ITM-CNR, I-87030 Rende (CS), Italy; Email: a.gugliuzza@itm.cnr.it

**Keywords:** membranes, valved-pores, photo-switching azo- and spiro-derivates, liquid crystals, polypeptides membranes, hydrogel membrane, light-driven devices, wettability, sensors, microfluidics

## Abstract

In recent years, stimuli responsive materials have gained significant attention in membrane separation processes due to their ability to change specific properties in response to small external stimuli, such as light, pH, temperature, ionic strength, pressure, magnetic field, antigen, chemical composition, and so on. In this review, we briefly report recent progresses in light-driven materials and membranes. Photo-switching mechanisms, valved-membrane fabrication and light-driven properties are examined. Advances and perspectives of light responsive polymer membranes in biotechnology, chemistry and biology areas are discussed.

## 1. Introduction

Smart membranes are in high demand in many advanced fields of biotechnology, including drug delivery, biosensors, microfluidics, light-powered molecular machines, molecular shuttles and data storage. Responsive polymer membranes are systems that sense the changes in their environment as a stimulus and make a desired response. In all cases, either a controlled porosity or a texture and chemical composition is coupled with adaptive properties, such as pH-, ionic strength-, thermo-, light-, electric-, and magnetic-response and molecular-recognition. The category of responsive membranes hosting reactive functions is large and includes flat and hollow-fiber membranes, nanocomposites layers, free-standing hydrogels, micro-capsules, switchable interfaces, core-shell structures, *etc.* A number of excellent books and reviews on membrane model systems have been published both on theoretical and applicative aspects [1,2,3], and many studies involving photo-induced effects on polymers have appeared [4,5]. The present review summarizes the recent developments in methods for the preparation of smart membranes and the mechanisms of their response to external stimuli with a particular attention to the behavior of light responsive polymer membranes. 

## 2. Photo-Switching Compounds and Mechanisms

According to the subdivision of Kinoshita [3], typical photo-reactive guests in polymers are azobenzene, triphenylmethane and spiropyran groups, which have been entrapped [6,7,8], cross-linked [9,10], and introduced as a side chain or part of the main chain [11,12,13,14,15] in polymer matrices. Special mention is deserved to photo-responsive polypeptide membranes.

### 2.1. Azobenzene-Based Systems

**Figure 1:**
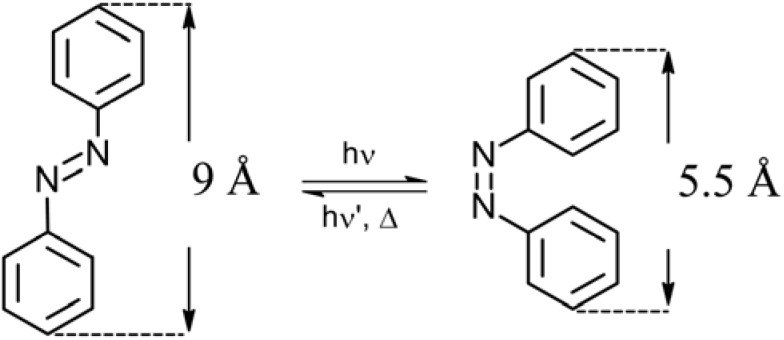
Photo-induced structure change in azobenzene molecule.

Azobenzene, AZB, derivatives are very attractive systems due to their easy *trans* → *cis* isomerization. Azobenzene groups can undergo an isomerization from a *trans* form to a *cis* form upon UV irradiation. The *trans* form is generally the more stable (energy gap ≈ 50 kJ/mol). AZBs have an intense π–π* band in the UV region, and a weak n–π* band in the visible region. The reaction is reversible by heat or irradiation with visible light, as shown in Figure 1. It is known that azobenzenes reversibly change their geometry from a planar one to non planar upon irradiation with a drastic decrease in the distance between the *para* carbon atoms from 9.9 Ǻ to 5.5 Ǻ and a corresponding increase in the dipole moment from 0.5 D to 3.1 D. As different geometries, polarities and electrical properties affect the two isomers, several functions can be photo-controlled including membrane dimensions, membrane potential, adsorption, solubility of polymer, wettability, swelling, enzyme activity, sol-gel transition of polymer, permeability, ion permeability, ion binding, photo-mechanical cycle, *etc*.

### 2.2. Triphenylmethane-Based Systems

**Figure 2:**
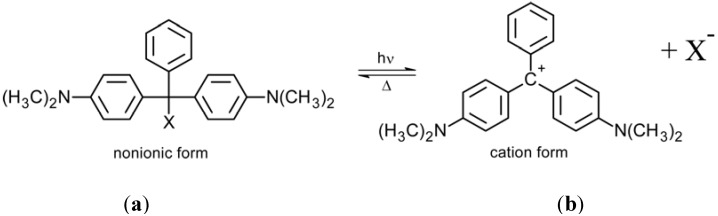
Photo-induced structure change of a triphenylmethane molecule: (**a**) non-ionic form, and (**b**) cation form.

Under UV irradiation, triphenylmethane groups (transparent form, Figure 2a) dissociate into ion pairs with the formation of triphenylmethyl cations (colored form, Figure 2b). The reaction is thermally reversible. Triphenylmethane leuco derivatives have been used in order to get photo-induced charges in polymer side chains, to obtain photo-induced changes in hydrophobicity, dilation and permeation through membranes. 

### 2.3. Spiropyran-Based Systems

**Figure 3:**
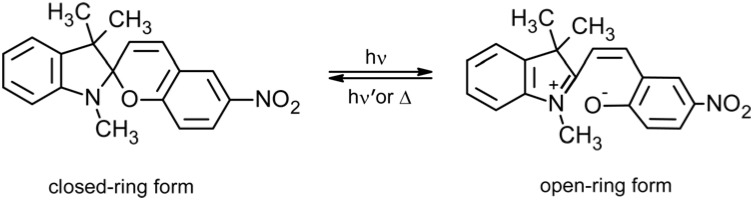
Photo-induced structure change of spiropyran.

Spiropyran, SP, is a well-known photo-chromic group that undergoes a heterocyclic ring cleavage at the C–O spiro bond to form a planar and highly conjugated chromophore that absorbs strongly in the visible region, this being the merocyanine, MC, isomer (Figure 3) [16,17]. The open-ring form returns to the initial close-ring form either by a thermal or photo-chemical process. Spiropyran derivatives can be entrapped, cross-linked, and introduced as side chain or part of the main chain in polymer matrices in order to gain a photo-regulation of membrane potentials. The MC isomer also binds H^+^ [18], divalent metal ions [19], DNA [20] and amino acids [21] resulting in a shift in the absorbance spectrum, and a corresponding colour change. This binding is photo-reversible under the right conditions, as illumination with white or green light decouples the guest from the MC, which then reverts to the SP. Hence spatial and temporal photo-controlled (and colorimetric reporting) uptake and release of guest molecules is, for instance, possible using spiropyran-functionalized materials [22].

### 2.4. Photo-Pesponsive Liquid Crystal Devices

Thermotropic liquid crystals are well known anisotropic materials, which deserve particular mention for their applications as electro-optical devices. Nematics are liquid crystals characterized by a long orientational order along a preferential direction, called director, which give them optical, dielectric and magnetic anisotropies. Accordingly to the direction of alignment of the director in the liquid crystal cell, one can distinguish homeotropic alignment, when the director is perpendicular to the cell substrates, planar or homogeneous alignment when the director is parallel to the cell substrates, or random when no preferential alignment is shown by the director. It should be mentioned that the nematic phases lose their anisotropic properties on heating above a characteristic temperature, called clearing temperature, or by increasing doping with molecules, which destabilize the attractive interactions acting between liquid crystalline molecules, or by photo-isomerization of photo-responsive additives. In fact, it has been shown that *cis* isomer induces more disorder than the *trans* one allowing the opportunity of reversible nematic → isotropic phase transition in liquid crystal by simple UV irradiation [23].

**Figure 4:**
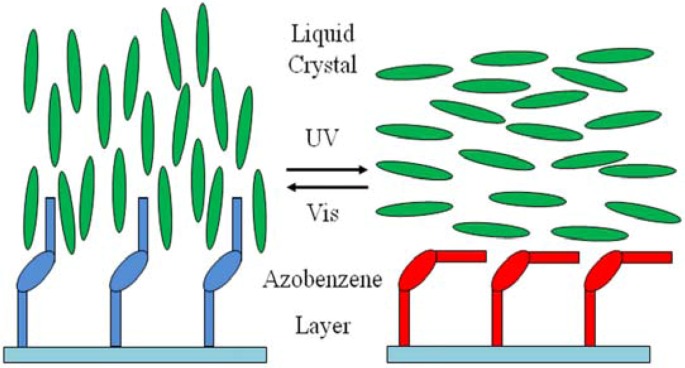
Photo-controlled alignment in liquid crystals.

It is usually important to align the liquid crystal director along a well defined direction, and, generally, the anisotropic properties of liquid crystals allow them to be easily aligned by an electric or magnetic field, by mechanical action, or by an alignment agent coated on the cell substrates (surfactants, lecithins, polyimides, *etc*.) [23]. Such initial alignment is changed by application of an external field like an electric or magnetic one, which exerts an electric or magnetic torque on the liquid crystal director. A different method of reversible liquid crystal alignment regulation has been reported for the first time by Ichimura and coworkers [24,25,26] using a photo-chemical reaction taking place on the substrate surfaces. In the particular case considered by Ichimura *et al.*, the *trans* → *cis* photo-isomerization of photo-reactive units (UV light at 366 nm for some tens of seconds) is able to change the director alignment from a homeotropic to a planar state, while the *cis* → *trans* back-isomerization (Vis light at 436 nm for some tens of seconds) allows the reverse transition. The authors treated the liquid crystal cell substrates with photo-chromic layers prepared by several techniques, including silylation, Michael addition, spin coating of polymers, Langmuir-Blodgett films, and always found a reversible homeotropic to planar transition in nematic liquid crystal cells. As reported previously [24,25,26], the authors synthesized also a series of poly(vinyl alcohol) derivatives having AZB side chains with different lengths and investigated the photo-response of liquid crystal alignment as a function of molecular structure of the Langmuir-Blodgett films, number of deposited layers, 2D density of azobenzene units, deposition and irradiation method. They found that one Langmuir-Blodgett monolayer was sufficient to induce liquid crystal alignment changes if the AZB unit was linked to poly (vinyl alcohol) backbone by a sufficient long spacer. Photo-responsive cells were obtained using both vertical dipping and horizontal lifting deposition methods. The irradiation with linearly polarized UV light induced a reorientation of liquid crystal director along a direction perpendicular to the polarization plane and dependent on the spacer length and number of deposited layers (Figure 4). The response times could be reduced by using high intensity sources.

An important photo-effect in aligned nematic liquid crystals is the optical Freedericksz transition, *i.e.*, the director reorientation by the electric field of the propagating wave. The effect can be enhanced in the presence of dyes (Janossy effect) and the optical Freedericksz threshold voltages can be lowered by two order of magnitude. Both effects have found interesting counterparts in polymer systems with liquid crystalline pendants [27,28,29,30,31].

Another interesting effect is the photo-control of layer thickness in lamellar liquid crystals, such as smectic A. The experimental results of Folks *et al.* have shown that the *cis-trans* isomerization of dispersed dyes can decrease the smectic layers’ spacing. Lansac and coworkers have confirmed by computer simulations that the positional ordering of azo-solutes in a smectic phase depends strongly on their photo-chemical state [32,33].

Voloschenko and Lavrentovich have investigated the light-induced phase separation phenomena in dye-doped liquid crystals and Chandran *et al.* have recently obtained the photo-control of colloidal-liquid crystal interfacial properties in solid colloids that were surface functionalized with photo-responsive azobenzene units and dispersed in liquid crystals [34,35].

**Figure 5:**
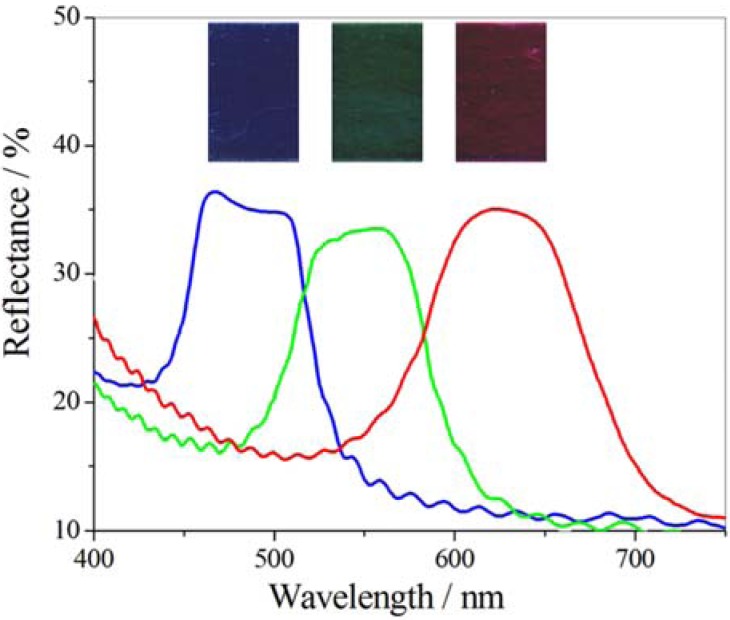
Progressive degradation of photo-sensitive chiral agent by UV light and corresponding reflected colors.

Another class of liquid crystals is cholesterics. They have a helical structure which selectively reflects a light wavelength associated to the helical pitch. Several electro-optical cells have been designed in order to have multicolor devices, and recently, a new cholesteric film, obtained by oriented cholesteric liquid crystalline molecules confined in elliptic droplets dispersed in a monomer matrix, has been proposed [36]. The cholesteric mixture consists of a nematic liquid crystal, three chiral agents including the photo-tunable material (S)-1,1’-bis(2-naphthyl)-4-(4’-pentyloxyphenyl) benzoate, a photo-polymerizable monomer and an UV initiator. The fluid mixtures were subjected, first, to a thermally induced phase separation in order to confine liquid crystalline components in elliptic droplets, then to a fast polymerization of the monomer matrix. The reflected wavelength is centered at 480 nm, but after UV irradiation with low energy density the peak moves to 550 nm and then, for higher energy density to 630 nm, giving rise to different reflected color, Figure 5. The change in the reflected peak is due to the progressive degradation of photo-sensitive chiral agent, which changes the helical twist power of cholesteric liquid crystal.

### 2.5. Photo-Responsive Polypeptide-Based Systems

In the last decades, much interest has risen in systems that could change the permeability across a membrane by a conformational change of polypeptides induced by photo-isomerization of the chromophore attached to the polypeptide.

As the photo-isomerization of the azobenzene groups from the *trans*- to the *cis*-form involves changes both in their geometry and polarity, photo-responsive polypeptide-based systems can undergo several conformational changes upon light irradiation including α-helix → random coil and vice versa, left-handed α-helix → right-handed α-helix, β-form → random coil or α-helix. These changes are dependent on the solvent composition and the amount of AZB incorporated in the system. In particular, Kinoshita *et al.* [37,38] have reported their investigations on a poly(L-glutamic acid) membrane, PGA, containing 14.0 mol% azobenzene moieties in the PGA side chains and found, upon UV irradiation, an increase in water content of membrane as a consequence of the polarity changes of AZB groups. Changes both in membrane potential and cross-membrane conductance have also been observed as a consequence of the acceleration in the dissociation of L-glutamic acid groups. The change in the potential membranes indicates an increase in the membrane negative charge, whereas the variation in the conductance could be attributed to the increase of ion diffusion through the membrane. The photo-induced nature of the observed changes has been confirmed by the absorbance at 350 nm of the AZB moieties, which was correlated with the variation of membrane potential and permeability.

The introduction of azobenzenesulfonate moieties (14.1 mol%) into PGA caused also a non-reversible α-helix → random coil transition in PGA after UV irradiation [39,40,41]. The membrane potential in azobenzenesulfonate doped PGA membranes shows a decreasing behavior after UV exposure. On the basis of these results the same system has been adsorbed onto a porous support (Millipore filter with a pore size of 0.1 μm) and the photo-induced permeation change has been investigated. Photo-irradiation induced rapid changes in the water flux through the membrane due to the photo-induced conformational changes (α-helix → random coil, pH 4.0) resulting from the *trans* → *cis* isomerization of the azobenzenesulfonate moieties. Also it has been shown that the photo-induced conformational changes are irreversible, *i.e.*, the *cis* → *trans* back transition of the azobenzenesulfonate moieties was not able to restore the original α-helix membrane structure.

A reversible photo-controlled (both in the conformation and functions) membrane was obtained by introduction of 10 mol% of pararosaniline leucohydroxide moieties in a poly(L-glutamic) membrane (2–3 μm thick) [42,43,44]. The conformation change was induced by a pH increase due to the photo-dissociation of OH^-^ ions from pararosaniline groups. Such system is characterized by a α-helix ordered structure around near weak alkaline pHs (7.5 < pH < 10.5), whereas it is in a random coil conformation at low (pH < 7.5) and high (pH > 10.5) pHs. From circular dichroism measurements it was established that the α-helix structure is lost at low and high pH values due to the amphoteric nature of membrane side chains. In fact, the L-glutamic acid moieties are negatively ionized at high pHs and the pararosaniline groups (pKa = 7.6) are positively charged at low pHs, and, consequently, far from weak alkaline pHs large repulsion forces originate from side chains bearing same sign charges. In the pH range 7.5–10.5 the polymer can form a α-helix structure as the charged side chains neutralized each other.

**Figure 6:**
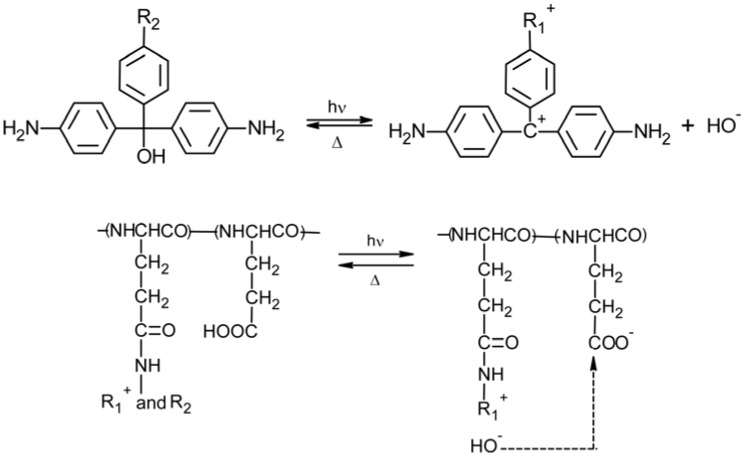
Photo-dissociation of pararosaniline groups and acid dissociation of L-glutamic acid groups.

Depending on pH, UV irradiation could induce either an α-helix → random coil or random coil → α-helix transition in the membrane due to the increase of hydroxide ion concentration from the photo-dissociation of pararosaniline groups according to the reaction and to the acid dissociation of L-glutamic acid groups (Figure 6) with a consequent change in the pH value and in the conformational state of the membrane. 

Synchronous changes in the permeability coefficient of styrene glycol and in the degree of hydration of a PGA membrane containing 15.5 mol% pararosaniline groups were respectively around +50% and +3% after ten minutes of UV irradiation at pH 8.6, *i.e.*, the increase of the permeability could be correlated to the photo-induced increase in membrane hydrophobicity. Both the changes were reversible after a dark adaptation of 100 minutes, respectively. 

A further improvement in the photo-responsiveness of PGA membranes was obtained by using triarylmethane leucocyanide groups (pararosaniline leucocyanide moieties) in the side chains [45]. Upon UV irradiation, pararosaniline leucocyanide groups photo-dissociate and the electrostatic repulsion among cationic side chains induces the α-helix → random coil transition of PGA membranes with 38.3% parosaniline leucocyanide moieties at pH 5.3. This transition results in an increase of the permeability coefficient of KCl across the above mentioned membrane by a factor 16, while the change in the degree of membrane hydration was around 300% on a 30 minutes light irradiation. A full recovery of the initial values of both permeability and hydration was obtained after a dark adaptation of 15 hours. In this case the pH was not changed as the photo-irradiation determines just the dissociation of triarylmethane leucocyanide in triarylmethane cations and CN^−^ ions, thus one of the main factors for the measured permeability coefficient is the increase in the free volume of the membrane due to the photo-induced swelling.

An example of photo-regulated permeability across a membrane by photo-induced conformational change of the polypeptide chains without any concomitant change in electrostatic repulsion along the polypeptide chain was achieved by Aoyama *et al.* [46,47]. They synthesized a new polyvinyl/polypeptide graft copolymer composed of a photo-responsive copolypeptide branch from β-p-phenylazobenzyl L-aspartate and β-benzyl L-aspartate attached to a poly(butyl methacrylate) backbone. They found that the permeation rates (in % h^−1^) of some polar and non-polar solvents across the membrane (30 μm thick) increased with UV irradiation and were suppressed on irradiation with visible or with dark adaptation. In particular, the permeation rates increased by a factor ranging from 1.5 to 3.7 with a maximum value for the permeation rate of mandelic acid across the membrane immersed in trimethyl phosphate, which was 5.7 times higher than that in the dark. The photo-induced permeability changes were correlated to the conformational change of the polypeptide chains in the membrane as confirmed by circular dichroism, CD, spectra. In fact, the UV irradiation changed the sign of the CD band within 15 min (the positive CD band at 215 nm change in a negative CD band centered at 222 nm), indicating the inversion of the helix sense of the polypeptide from left-handed to right-handed. The original CD profile could be recovered by irradiation (5 min) with visible light or by dark adaptation (5 days).

The introduction of photo-responsive units in supramolecular systems may be important for their practical applications. Higuchi and Kinoshita [48,49] synthesized and characterized a photo-responsive amphiphilic polypeptide formed by two amphiphilic α-helical polypeptides, poly[(γ-methyl L-glutamate)-co-(L-glutamic acid)], bringing an AZB moiety which was responsible of changes of aggregate structure in aqueous solution upon irradiation with UV light. In particular, when the polypeptides were incorporated into lipid membranes, they formed transmembrane bundle in the dark, acting as an ion-permeable pore through the membrane. The *trans* → *cis* photo-isomerization of the AZB linkers induced reversible changes in the pore structure allowing the photo-regulation of ion-transport across the membrane. The polypeptides self associated in water to form globular aggregates with an average diameter of 10 nm in dark conditions. Over a certain light intensity threshold the *trans* → *cis* photo-isomerization of the AZB linkers induced a bending of polypeptide rod and a consequent structure disaggregation of globular aggregates in smaller and disordered ones (around 3 nm).

## 3. Light-Switching Functions

Light can be considered as a clean stimulus that allows remote control without physical contact or a mechanical apparatus. It is attractive because it enables one to change the geometry and dipole moment of photo-switching molecules causing macroscopic variations of molecularly organized structures by small perturbations. These changes can affect final properties such as wettability, permeability, charge, 3-D shape, color, binding, and alignment. A fine-tuning of these changes can be done through a series of sophisticated techniques listed in Table 1 [50].

**Table 1:** A list of some techniques used to monitor morphology and property changes due to photo-irradiation.

Technique	Property	Reference
UV–Vis spectroscopy	Isomerization	[51]
Ellipsometry	Variation of the average thickness of the sample in fair agreement with the calculated geometries of the molecules.	[52]
Surface plasmon resonance spectroscopy	Switching in real time under ambient conditions	[53]
Contact angle measurements	Switching wetting of the surfaces	[50]
Adsorption of molecules/particles from solution	Control of adsorption on surfaces	[50]
Atomic force microscopy	Switching in individual molecules	[52]
Kelvin probe measurements	Changes in the work function of functionalized surfaces	[54]
Measurements of electrical properties	Azobenzene switching controls electrical properties of SAMs	[50]
Electrochemical methods	Quantitative isomerization by cyclic voltammetry	[55]
Surface-enhanced Raman spectroscopy	Isomerization on the surface	[56]

### 3.1. Light-Driven Wettability

The wettability is a surface property detectable by contact angle, CA, measurements. The apparent contact angle is a result of attractive and repulsive interactions at the solid/liquid/gas interfaces, expressed by polar and non-polar contributions to the overall surface free energy [57]. Based on Young’s equation, hydrophilicity conventionally refers to CA of less than 90° on solid surfaces, while hydrophobicity refers to CA higher than 90°. However, this distinction appears to be too simplistic if one considers that the apparent contact angle depends on both the surface chemistry and morphology. Then, membranes prepared from chemically analogous compounds can exhibit different wettability depending on the degree of surface roughness. 

Considering light-driven systems, the variation of dipole moment associated to the *trans*-isomer in azo-switching compounds and to the mero form in spiropyran derivates can produce dramatic changes in surface free tension, resulting in enhanced surface wettability.

It has been observed that a variation of 10° can be induced on azo-functionalized smooth and flat surfaces by photo-isomerization [58]. Tylkowski *et al.* [59] proposed a study about the light-driven wettability of novel asymmetrical poly(vinyl alcohol)-co-ethylene membranes blended with azobenzene polymers. A reversible variation of wettability (*θ_E_ − θ_Z_ = 26°*) was estimated as a consequence of dramatic changes in surface roughness moving from isomer E to isomer Z. An increase in hydrophilicity was ascribed to the migration of azo polymer towards the top surface as well as to the conformational change in co-polymer caused by photo-irradiation.

**Figure 7:**
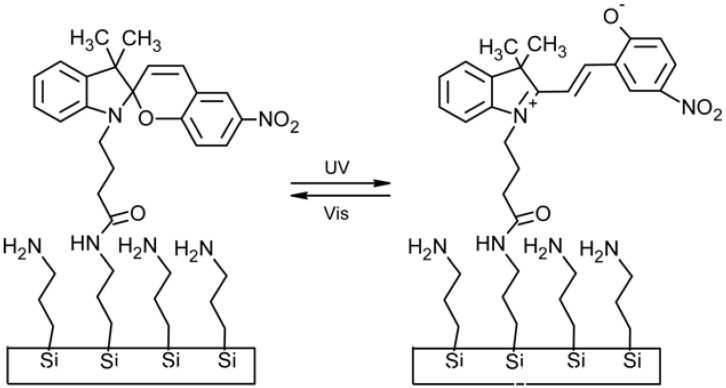
UV-Vis induced changes in wettability (Adapted from Vlassiouk [62]).

Polymers containing spiropyran moieties have been grafted onto membrane surfaces, causing reversible wettability-switching [60] and protein adhesion [61]. Similarly, Vlassiouk and coworkers [62] fabricated nanoporous alumina membranes bringing spiropyran-terminated linkers (Figure 7). 

Upon exposure to UV light, a reversible switching from non-polar to polar form produced higher wettability allowing water and ions to diffuse through the pores. In this case photo-chromic molecules acted as on/off valves via reversible wetting mechanisms. 

Also, photo-isomerization of diarylethene molecules has been demonstrated to direct the surface wettability moving from CA of 163° to CA of 120° [63].

Photo-switchable nanoporous multilayer films have been realized anchoring fluorinated AZB molecules, 7-[(trifluoromethoxyphenylazo)phenoxy]pentanoic acid, on a film prepared from poly(allylaminehydrochloride) and SiO_2_ via layer-by-layer techniques. Changes in dipole and surface roughness addressed the surface at hydrophilic and superhydrophilic zones using selective UV irradiation through an aluminum mask [64].

**Figure 8:**
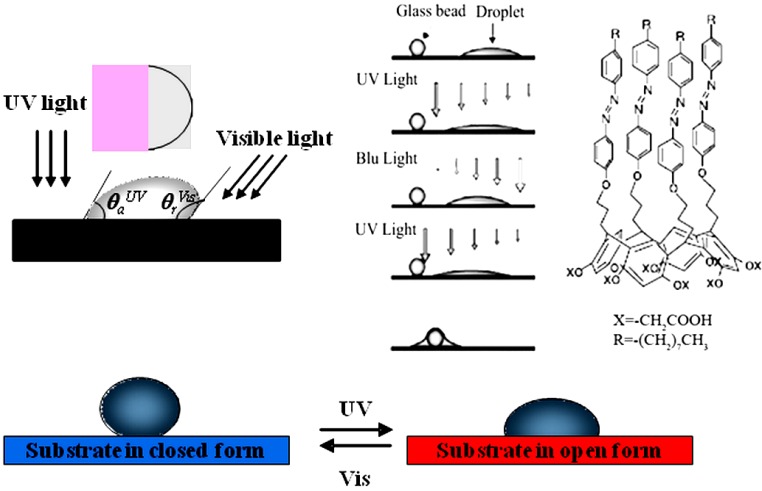
Photo-driven motion of individual liquid droplets (Adapted from [68]).

Switching wettability is an attractive issue with the construction of molecular shuttles [65]. Light-driven motion of individual droplets is a very striking item, if one considers that conventional fluid control components, such as valves and pumps, are not easily scalable to the small dimensions of the future fluidic chips [58,66,67]. Photo-isomerization of AZB-active surfaces has been demonstrated to shift millimeter liquid droplets along pathways under a gradient in surface free energy generated via imbalance of contact angles on both the edges of the droplets, Figure 8 [68].

However, surface hysteresis and surface defects could cause the liquid droplet to not move itself under spatially controlling UV/Visible irradiation. Light-driven motion of the droplet can be induced when the forces exceed the droplet pinning on the smooth surface. This needs the establishment of a gradient of wettability by irradiating one-half of the droplet with UV light and the other half with visible light (Figure 8). When the forces go over the hysteresis, the droplet will be pulled towards UV irradiation if the advancing contact angle of half droplet with *cis*-isomers (*θ*_adv_^UV^) is lower than the receding contact angle of half droplet with *trans*-isomers (*θ*_rec_^vis^).

This means that light-induced contact angles should be greater than contact angle hysteresis (Δ*θ* = *θ*_adv_ − *θ*_rec_) in the *trans* state [69]. In this respect, it is instructive to describe the equations that express the forces for the light-driven liquid motion. The unbalanced Young’s force for a section of the droplet of width dx is given by

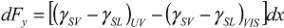
(1)
where *γ_SV_* and *γ_SL_* are the surface free energies of the solid-vapor and solid-liquid interfaces after UV or visible irradiation of the surface and *dx* is the thickness of the section of the droplet. If *θ_vis_* and *θ_UV_* represent the local contact angles at the edges on the two sides of the droplet, then, Equation 1 can be written as

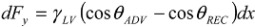
(2)
where *γ_LV_* is the surface free energy of the liquid-vapor interface. Therefore, the net force (*F_y_*) on the droplet can be expressed by the difference in the advancing and receding contact angles on the two sides of the droplet and by integrating Equation 2 over the width of the droplet (*w*).

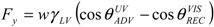
(3)

### 3.2. Light-Adaptative Membrane Gates

Valved-membranes are considered as smart artificial channels for selective and programmable mass transfer, including ions, liquids and gases. The possibility of programming molecular transport for ions, liquids, metals and gases in response to photo-irradiation opens the way to build up miniaturized nanodevices including (bio)sensors, autonomous drug-delivery systems, microfluidic valves and flow switches, ‘lab-on-a-chip’ systems, (bio)fuel cells and so on. Different successful approaches to realize light-driven membrane pores have been proposed over the years and hereafter are illustrated.

#### 3.2.1. Photo-Controlled Ion Permeation through Membranes Gates

**Figure 9:**
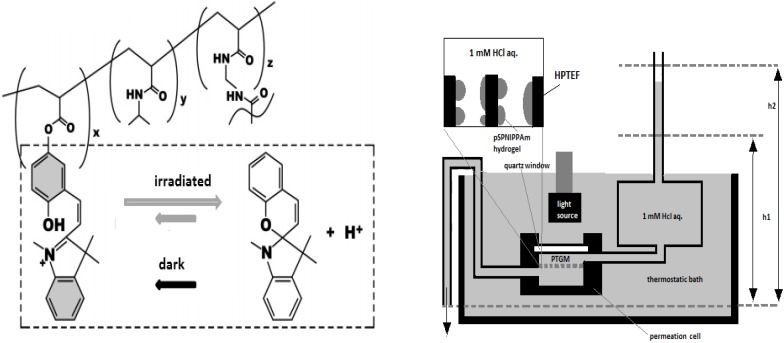
Light-controllable devices for microfluidics.

Poly(styrenesulfonate) and a polyacrylamide copolymer containing a photo-chromic chromophore, 2-nitro-4’-methoxyazobenzene, have been cast via layer-by-layer on porous alumina in order to form light-valved membranes [70]. In this case, an increase of 1.6 times in SO_4_^2−^ permeability has been observed under UV irradiation, suggesting a strong sensitivity of the pore size to the voluminous expansion and contraction of the azo compound. Similarly, photo- and thermo-responsive membrane gates have been realized by introducing a spirobenzopyran residue into N-isopropylacrylamide-based hydrogels [71]. The precision to regulate on a microscale the membrane allowed the construction of light controllable devices or microfluidics (Figure 9).

Alternatively, photo-responsive compounds have been covalently anchored to the walls of membrane pores in order to leave enough free volume to motional freedom and, then, to isomerize [72,73,74]. Also, AZB derivatives have been anchored inside magadiite, causing a reversible change in *d*-spacing of 0.6Å upon UV-Vis irradiation [75]. Hexagonal mesoporous silica has been doped in 1,2-bis(4-pyridyl)ethylene solutions [76]. In this case, switchable moieties have been preferred to layered polymers for realizing 3D nanocomposite hybrid structures through which mass transfer can be controllated easily.

Liu and coworkers [77] have proposed the synthesis of surfactant-directed self-assembly of a novel photo-responsive azobenzene-containing organosilane, 4-(3-triethoxysilylpropylureido) azobenzene, into an ordered, periodic silica framework to make photo-responsive nanocomposites. A variation of pore size of 6.8Å together with a significant change in the dipole moment (0–3D) suggested these hybrid systems to be promising ion-channel membranes. 

**Figure 10:**
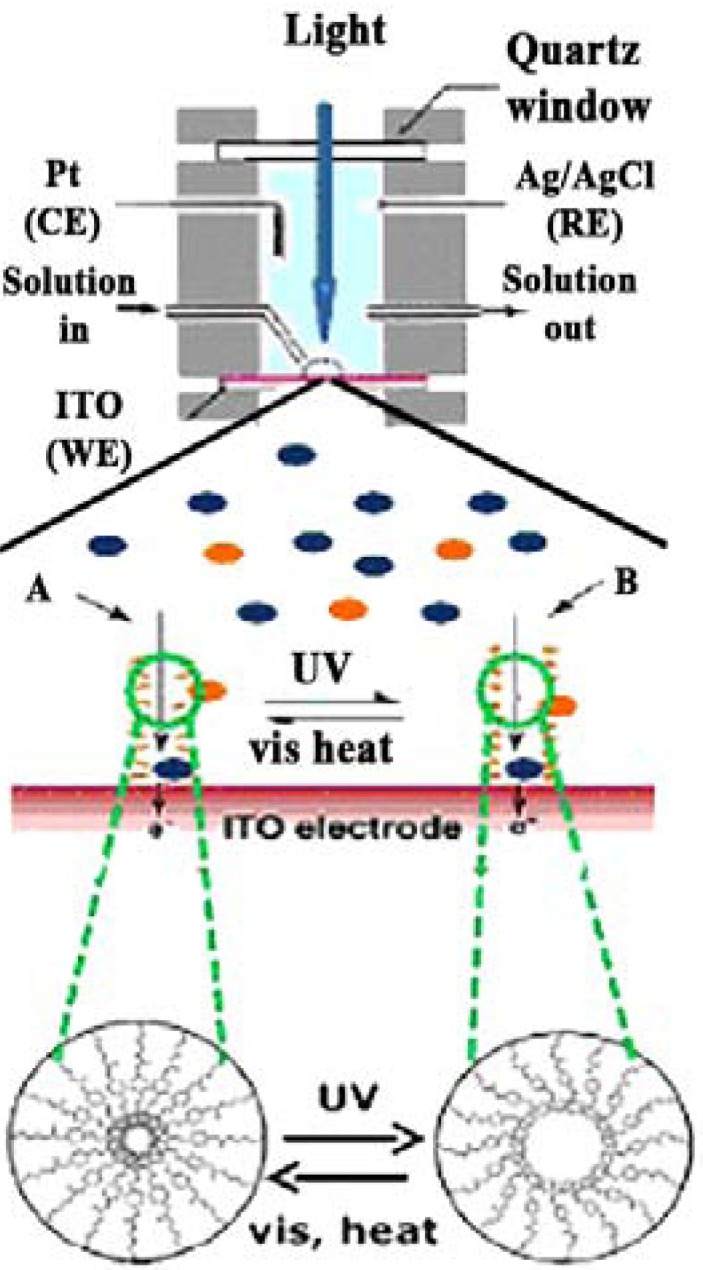
Photo-responsive nanocomposites proposed in [78].

Similar systems have been processed by using ferrocene dimethanol or ferrocene dimethanol diethylene glycol as a molecular probe [78]. In particular, the steady-state oxidative currents at constant potential was monitored for the reactions taking place on the working electrode surface (Figure 10).

**Figure 11:**
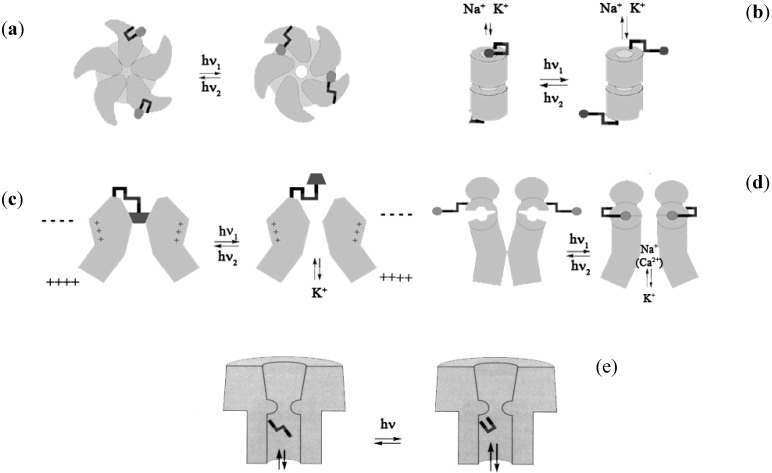
Light responsive ion channels mimicking “artificial sense”.

The build up of valved membranes is one of the most attractive issues also in the area of biotechnology, if one considers the possibility to command biological functions through artificial light-sensitive ligands. The literature refers to some chemical modifications to physiologically relevant ion channels that make them light-sensitive enabling them to work in an “artificial sense” [79]. Specifically, nicotinic acetylcholine receptor (Figure 11a), gramicidin A (Figure 11b), a voltage-gated potassium channel (Figure 11c), an ionotropic glutamate receptor (Figure 11d), and α-haemolysin (Figure 11e) have been considered for optical manipulation with a high degree of spatial and temporal control. In the first case, a light-gated nicotinic acetylcholine receptor uses a photo-switchable tethered agonist conjugated to the channel surface via an AZB linker such that the *trans* isomer activates the channel (Figure 11a). Figure 11b shows a light-gated gramicidin A using a photo-switchable tethered ammonium ion pore blocker conjugated to the C-terminal ethanolamide via AZB linker such that the *cis* isomer blocks the channel. In the light-gated potassium channel the photo-switchable tethered blocker is an ethyl ammonium ion pore conjugated to the channel surface via an azobenzene linker such that the *trans* isomer blocks the channel (Figure 11c). An example of light-gated ionotropic glutamate receptors is depicted in Figure 11d and uses a glutamate analogue conjugated to the channel surface via AZB linker such that the *cis* isomer activates the channel. Finally, the light-gated α-haemolysin is functionalized with a sulfonated azobenzene conjugated to the channel lumen such that the *trans* isomer impedes conductance more than the *cis* isomer.

In all cases a straightforward functionalization of the chromophore changes the wavelength sensitivity, affecting the thermal stability, extending the photo-conversion between isomers and reducing the photo-toxicity often associated with UV irradiation for biological systems. 

Sata *et al.* [80] reviewed studies on anion exchange membranes and electrodialysis methods to permeate specific anions through the membranes, also by photo-irradiation. 

**Figure 12:**
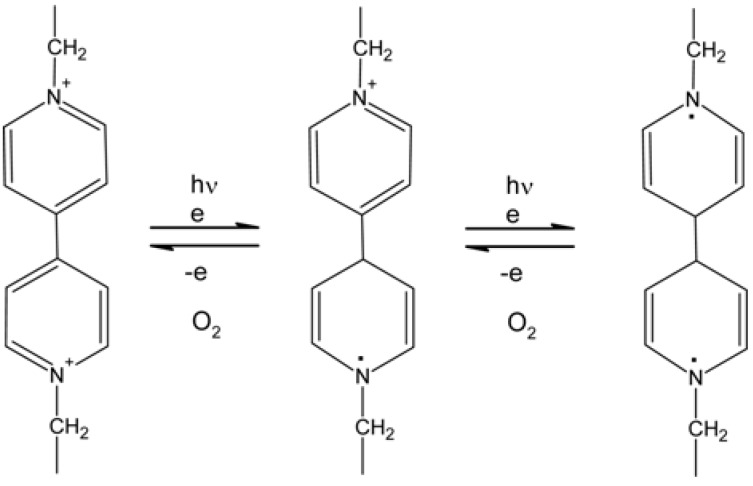
Reversible reduction and oxidation of a viologen moiety of the anion exchange membrane.

It is well known that viologen compounds such as 1,10-dimethyl-4,40-bipyridinium dichloride are reduced to monocation radical and then biradical by the irradiation of UV around 320 nm as shown in Figure 12. Namely, dication changes in monocation and then the monocation changes into uncharged compounds in a reversible way.

When a cross-linked copolymer membrane bringing chloromethyl groups reacts with4,40-bipyridine, pyridinium groups are introduced as anion exchange groups and cross-linking reaction occurs due to diamine.

When light from a xenon lamp is irradiated on this membrane in pure water, 0.50 N sodium chloride solution or other salt solutions, new absorbance peaks at 406 and 615 nm appear, which are based on the formation of monocation radical—the color of the membrane changes from slightly pale yellow to deep blue—and these peaks decrease by further irradiation, which means the formation of biradical [81,82]. On the other hand, the blue color of the membrane bleaches by diping it into non-degassed salt solution or exposure to air. Namely, dications, monocation radicals and biradicals are reversibly formed in the membrane by photo-irradiation or exposure to oxygen, which means that the amount of anion exchange groups can be controlled by photo-irradiation. The membrane should shrink or swell by photo-irradiation and then pore size of the membrane is expected to change.

Membrane having a viologen moiety as anion exchange groups showed the change of permselectivity of anions (as SO_4_^2−^ or Br^−^) when electrodialysis was carried out in the presence of photo-irradiation after the membrane had been previously irradiated for a given period [82]. In particular, the transport number of anions apparently decreases by photo-irradiation when compared with that of the membrane without irradiation. This is thought to be due to surface shrinking and decrease in hydrophilicity of the membrane, because of the decrease in anion exchange groups. 

The transport number between anions through the membrane having an AZB moiety is expected to change due to the *trans* → *cis* transformation of the moiety by photo-irradiation [83].

Recently, Byrne *et al.* [84] have synthesized two photo-responsive linear polymers of different molecular weights based on benzospiropyran, BSP, and polymethylmethacrylate, PMMA. As the merocyanine isomer, MC, of BSP has a phenolate site to which transition metal cations can bind (such as Cu^2+^ and Co^2+^) and this reversible process can be optically controlled, these systems have been used to bind transition metal ions. The binding of a transition metal ion requires two units of the MC isomer to form the most thermodynamically stable MC_2_–metal ion complex [85,86]. Fries *et al.* [87] recently published the synthesis of a chemo-responsive co-polymer of PMMA and spiropyran methyl methacrylate and demonstrated that different metal ions give rise to unique colorimetric responses that are dependent on the amount of spiropyran co-monomer contained in the polymer backbone. 

Zhou [72] described the metal chelation mechanism formation and disassociation as follows: BSP + MC + Cu^2+ ^→ MC_2_–Cu^2+^. The MC isomer reacts readily with the Cu^2+^ ions as it is thermally formed from BSP and this formation process is the rate-determining step in the reaction sequence, with the Cu^2+^ ion concentration having little effect on the reaction rate.

Byrne *et al.* analysed UV-Vis spectra of their BSP-PMMA after addition of Cu(NO_3_)_2_ solution and observed, after the formation of the corresponding complex, significant rearrangements of the polymer chains which affect the physical and chemical properties of the system. This process is completely photo-reversible, as when [MC-PMMA]_2_–Cu^2+^ is irradiated with white light the MC photo-isomerizes back to the closed BSP isomer, ejecting the Cu^2+^ ion.

#### 3.2.2. Photo-Controlled Organic Liquids Permeation through Membrane Gates

Porous membranes with polymer brushes have the advantages of mechanical strength and quick response to an external signal over the hydrogel membrane. In addition, the signal-responsiveness of permeation through a porous membrane is apparently opposite to that estimated through hydrogel membranes.

**Figure 13:**
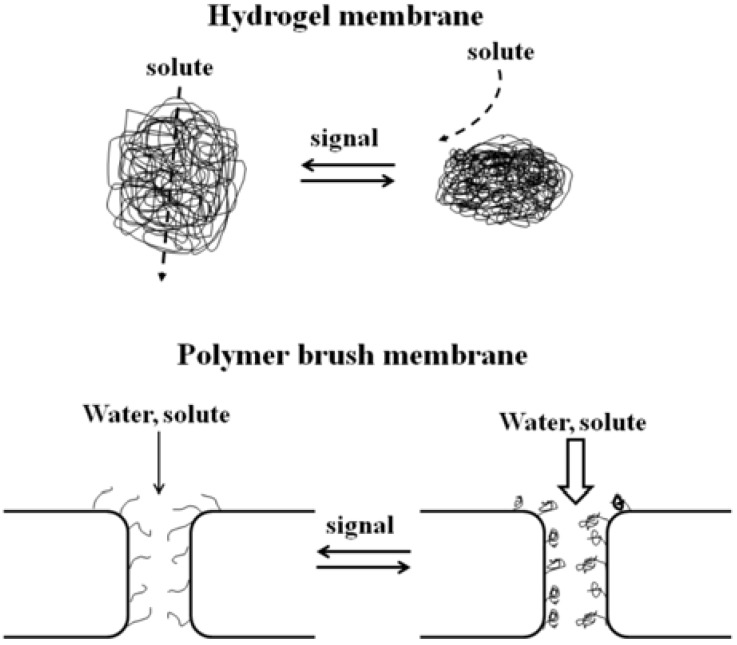
Regulation of water and solute permeation through a hydrogel membrane and a polymer brush-grafted porous membrane.

Hydrogels contract or expand in response to environmental conditions such as pH, temperature, photo-irradiation, solvent, or chemicals. The hydrogel swells in the ionized state to facilitate permeation of solutes, and deswells in the de-ionized state to suppress permeation (Figure 13, top). On the other hand, several polymer-brush membranes have been synthesized, in which pores turn open or close in response to environmental conditions. In particular there are several examples of photo-responsive porous membrane devices (Figure 13, bottom) [88].

Irie *et al.* [89,90] reported the solubility alteration, in cyclohexane, by photo-irradiation of polystyrene carrying spiropyran groups in the side chains. The permeation of organic liquids through the glass filter with and without surface grafts was studied with and without photo-irradiation. The glass filter with the copolymer grafts changed the permeability of toluene. On the contrary, the permeation of toluene through the same glass filter without grafts was not affected by photo-irradiation.

The permeability results were obviously related to the solubility of the graft copolymer. The absence of solubility change in *N*,*N*-dimethylformamide, DMF, led to the absence of permeability change by photo-irradiation. The copolymer graft in either the zwitterionic or the neutral state was soluble in DMF, so that the graft chains were well extended in DMF to close pores on the glass filter and the DMF permeation was irrespective of photo-irradiation. On the other hand, in a non-polar solvent such as toluene, the zwitterionic merocyanine form shows contracted graft chains and, consequently, open pores with an increased permeation of toluene. Because the neutral spiropyran form was soluble in toluene, the graft chains were extended to cover the pores of the glass filter, thus reducing the permeation of toluene in this case. 

Recently, Balazs *et al.* [91] showed by theoretical and computational modeling that the application of an external gradient in light intensity causes the dynamic reconstruction of a three-component membrane. In particular, they found that the photo-sensitive domains preferentially reorient along the gradient direction. They also found that this imposed gradient effectively controls the transport of a non-reactive component within the membrane. This non-reactive component migrates along the gradient intensity towards the higher values of intensity and the speed of this migration is defined by the actual value of the gradient.

#### 3.2.3. Permselectivity of Gases through Photo-Switching Membrane Gates

Weh *et al.* [92] observed the permeation characteristics of a membrane made from AZB in a polymethacrylate matrix. The photo-isomerization of AZB molecules causes changes in their molecular size and geometry as well as in their dipole moment. It can be expected, therefore, that the transport behavior of gases through such a membrane can be influenced.

It is known that bilayer membranes, which contain AZBs, can change their ion permeability by irradiation [93]. Balasubramanian *et al.* [94] reported the photo-induced variation of the electric resistance of a membrane, which consists of AZB containing water/oil microemulsions. By *trans*/*cis* switching of AZB in Langmuir-Blodgett films, the electric conductivity can be changed [95].

A photo-induced change of the potential and ion permeability in a polyvinyl chloride membrane, which contains AZB modified crown ethers, has been described by Anzai *et al.* [96].

Weh *et al.* [92] prepared polymethacrylate membranes, which contained AZB, as thin films supported on porous ceramic carriers. They measured the flux rates and *trans*/*cis* permselectivities for hydrogen, methane, n-butane, sulfur hexafluoride, and methanol. For a polymethacrylate membrane, which contains chemically bound AZB, characteristic changes of the permeation properties have been found by photo-chemical *trans*/*cis* switching of the AZB.

Among the three types of polymer membranes tested, only the membrane with AZB chemically bound to the side chain of the polymethacrylate showed a modified permeation behavior for different gases and methanol vapor when the AZB was photo-chemically switched into the *trans* or *cis* state. Due to the switching effect of the mass transport, this type of membrane acts like a microvalve. Possible applications of this membrane could be in microreactors for the controlled dosing of the educts or for the light controlled removal of products.

## 4. Photo-Responsive Membranes: Classes of Adaptive Materials and Their Applications

### 4.1. Photo-Responsive Polyelectrolyte Multilayer Membranes

Polyelectrolyte multilayer membranes present an interesting suitability for gas/salt separations [97], pervaporation from water/organic solvent mixtures [98,99,100], and reverse osmosis membranes [101,102]. Recently, there has been an increasing interest for this type of membranes also as gating membranes [103,104,105,106,107,108,109] considering that a current challenge is the construction of reversible valved-membranes, through which ion flow [110], capture and release of molecular targets [111] and energy transfer can externally be triggered.

**Figure 14:**
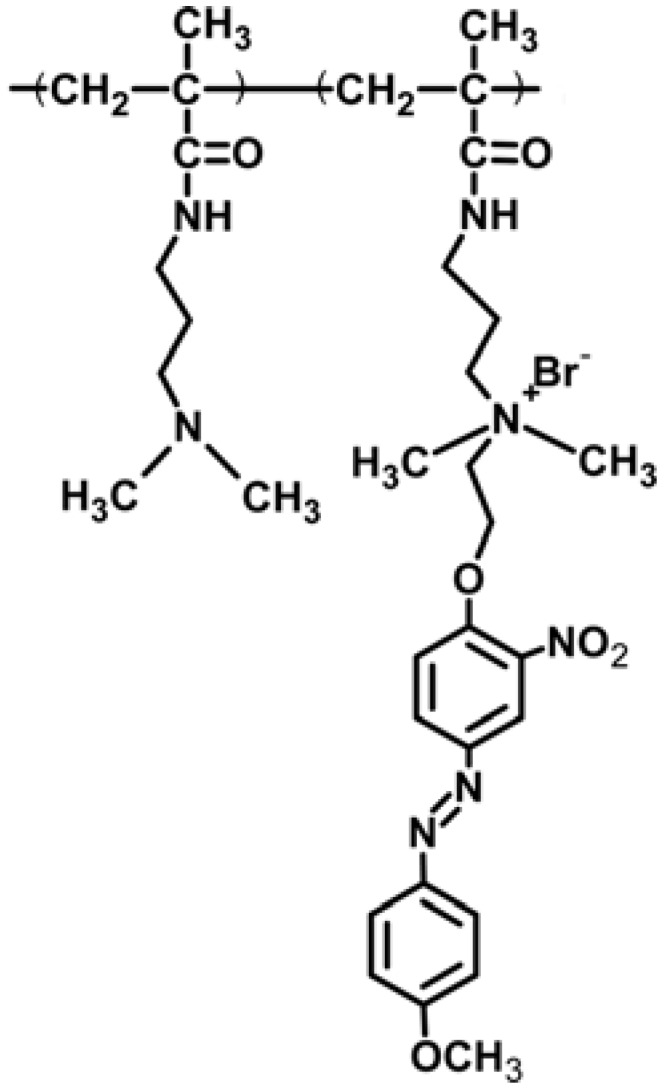
Polyacrylamide copolymer containing 2-nitro-4’-methoxyazobenzene, PNA, used in [70].

Kumar *et al.* have prepared photo-responsive multilayered membranes on a porous alumina support using layer-by-layer deposition of a negatively charged poly(styrenesulfonate) and positively charged polyacrylamide copolymer containing 2-nitro-4’-methoxyazobenzene (PNA, Figure 14), since efficient photo-chemical control of ion flow was expected through them [70].

Nanoporous alumina substrates were also used to fabricate a photo-responsive polyelectrolyte membrane by using spin-coating electrostatic self-assembly, a rapid technique which allows a precise control of the film thickness on a solid substrate [70]. Unblocked free pores (with a size of ~0.02 µm) were formed on bottom part of poly(styrenesulfonate)/PNA bilayers, whereas the top surface of the porous alumina membrane was completely covered with the pinhole-free smooth film.

UV irradiation (λ = 360 nm, P = 2 mW/cm^2^) on the polyelectrolyte membrane induced *trans* → *cis* isomerization of the nitroazobenzene species and enlarged pores inside the membrane as a result of the *cis* bent molecular structure, thus making sulfate and chloride ion transport easier. Subsequent exposure to visible light (λ = 450 nm, P = 100 mW/cm^2^) restored the original dense state of the membrane matrix, which in turn restrains the ion flow. 

The permeability of the bulky SO_4_^2–^ ions was more sensitive to the channel sizes. The ion-permeation rate of Cl^–^ ions increased only 1.2 times after UV irradiation of the polyelectrolyte membrane, whereas the one of the SO_4_^2–^ increased about 1.6 times.

### 4.2. Photo-Responsive Polymer-Grafted Porous Membrane

The photo-isomerization of photo-chromic groups incorporated into polymeric systems allows light-induced variations in polymer structure, conformation, and solvation because of the altered interactions between photo-chromic groups and polymer chains, solvent molecules, or polymer side groups. Thus, photo-reversible controlled solution viscosity [112], colloidal stability [113,114,115], surface wettability [116,117], solvent permeability [118,119] are some of the potential applications.

**Figure 15:**
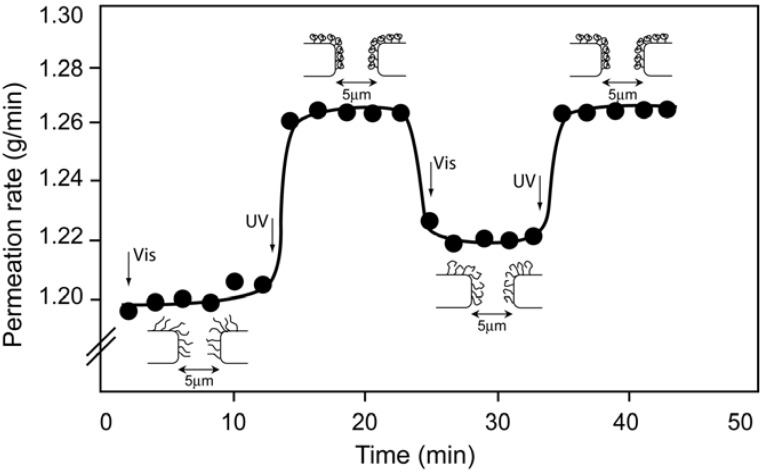
Permeation rate of toluene with a naive representation of copolymer grafts of [118].

Park *et al.* grafted a spirobenzopyran methylmethacrylate copolymer (SPMMA/MMA) on a porous glass membrane filter (nominal pore size = 5 µm) to control permeation of organic liquid by photo-irradiation [118]. After graft copolymerization, the surface wettability resulted altered, but no significant difference of wettabilities of graft-polymerized membrane was observed before and after UV-light irradiation. The permeation of *N*,*N*-dimethylformamide (DMF) through the grafted glass membrane did not depend on photo-irradiation. The permeability is obviously related to the solubility of the graft copolymer. The absence of a permeability change by photo-irradiation was attributed to the absence of solubility change of SPMMA/MMA copolymer in DMF by UV/Vis irradiation. The copolymer grafts in either the zwitterionic or neutral states of the pendant spirobenzopyran groups were soluble in DMF, *i.e.*, the graft chains were well extended in DMF to close the pores of the glass filter with unchanged DMF permeation irrespective of photo-irradiation. On the contrary, a variation in the toluene permeation was observed in response to photo-irradiation. As the zwitterionic merocyanine form of the copolymer grafts is not soluble in toluene, the graft chains were in a bend state, thus increasing the pores on surfaces of glass filter and then the permeation of toluene (Figure 15).

**Figure 16:**
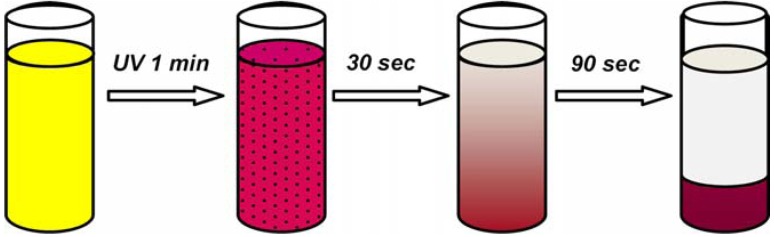
UV-induced aggregation and sedimentation of colloidal silica nanoparticles with spiropyran brushes.

Colloidal silica particles modified with end-grafted spiropyran-co-methylmethacrylate polymer brushes show also light responsive aggregation behavior, whereas surfaces exhibit photo-actuated changes in wetting properties [115]. These core-shell silica colloids form stable suspensions in toluene when the spirobenzopyran pendants are in their closed, non-polar form. After exposition to UV irradiation (λ = 366 µm), a rapid aggregation and sedimentation follows (Figure 16) due to UV-induced photo-switching to the open, polar merocyanine isomer. Because the SP ring-opening reaction is highly sensitive to the local environment and interactions with solvent molecules, core-shell silica particles dispersion stability depends on solvent polarity. An appreciable UV enhancement of sedimentation rate was recorded for tetrahydrofuran, o-xylene, benzene, and toluene 0.5% v/v suspensions of 927 nm silica spheres. In particular, sedimentation was 1.1 times faster in tetrahydrofuran, 6 times faster in o-xylene, 16 times faster in benzene, and 335 faster in toluene. The largest photo-induced aggregation effect in a given solvent can be achieved through the optimization of SP content, e.g., ~20 mol% for toluene.

SP to MC conversion caused a significant attraction between particles suspended in a poor-solvent medium, resulting in a UV-induced flocculation as a consequence of the change in particle surface polarity. The sedimentation may be associated to interparticle association of MC and SP moieties into dimers, (SP*_n_*MC)*_m_*, and MC_n_ intrachain complexes formed as products of photo-induced aggregation in SP(x)-MMA copolymers [120]. MC_n_ complexes are organized into stack structures having an antiparallel orientation of molecular dipoles that result in the appearance of an additional shoulder (λ = 545–550 nm) in UV/Vis spectra, which are generally characterized by a main peak around 578–598 nm [115,120]. The UV-induced flocculation process is fully reversible after visible light exposure (λ > 450 nm) and agitation (short sonication for less than 1 min or vigorous shaking). 

The rheological response of SP(20)-MMA-coated colloids was investigated upon alternating UV/Vis light irradiation. The viscosity of the ~30% v/v toluene suspension of 927 nm core silica particles (with 25 nm thick SP(20)-MMA brushes) was reversibly modulated by ~50% through successive irradiation with UV and visible light. The first UV/Vis treatment yielded slightly larger response than subsequent cycles, probably due to the difference between the initial equilibrium network structure and the one formed during shearing [115]. The UV-induced aggregation exhibits first-order kinetics and is significantly faster than visible-induced redispersion. This latter depends on combination of two relaxation processes with time constants differing by a factor of ~10 and suggests the formation of interparticle bonds with varying strength. The SP(20)-MMA-coated colloidal particulate dispersion viscosity increases as a consequence of photo-induced attractive interparticle interactions. 

**Figure 17:**
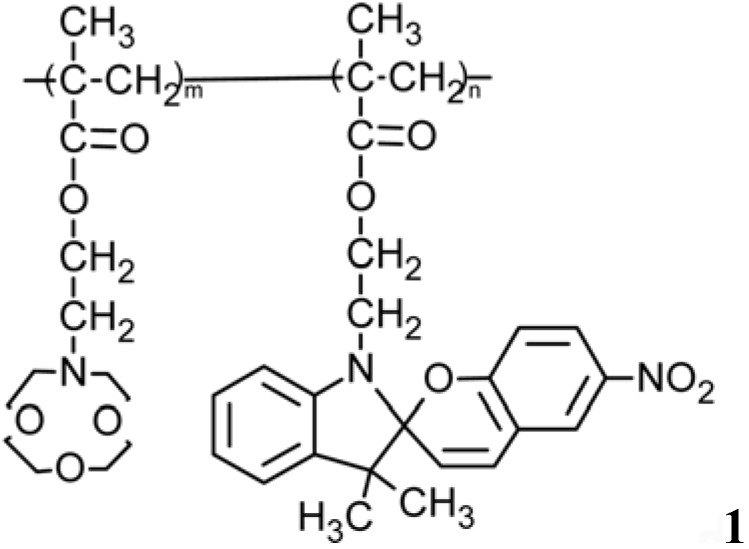
Chemical formula of crown ether–spirobenzopyrans copolymer of [121].

Kimura *et al.* applied a crown ether–spirobenzopyrans copolymer of methacrylate (m:n = 1:1), Figure 17, to a macroporous membrane in order to photo-control solvent permeation [121,122].

In copolymer **1** the isomerization induced by UV light irradiation of the spirobenzopyran moiety to the corresponding more polar zwitterionic form may cause an appreciable rheology change due to an increased merocyanine association. Thus, photo-isomerization brings polymer chain contraction/extension and interpolymer crosslinking. A blue shift of the merocyanine absorption indicates the formation of H-type aggregates [123]. UV-light irradiation on macroporous (pore size 10–100 µm) polyethylene membranes coated by the crown ether-spirobenzopyran copolymer leads to a significant decrease of the permeation rate of hexane through the membrane, due to increased hydrophilicity of its surface. 

When visible light is irradiated on crown ether-spiropyran copolymer coated polyethylene membrane, the initial permeation rate value is regained by isomerization back to the electrically neutral spiropyran form. Conversely, the permeation of ethanol through the membrane is decreased by visible light due to a reduction in the apparent membrane pore size induced by the polymer chain extension and increased by UV light. There was no photo-response in the solvent permeation with uncoated polyethylene membranes, while similar photo-responses in the permeation rate of non-polar and polar solvents were attained with a sintered glass filter modified chemically by both silane-coupling reagents containing distinct crown ether and spirobenzopyran moieties [122].

**Figure 18:**
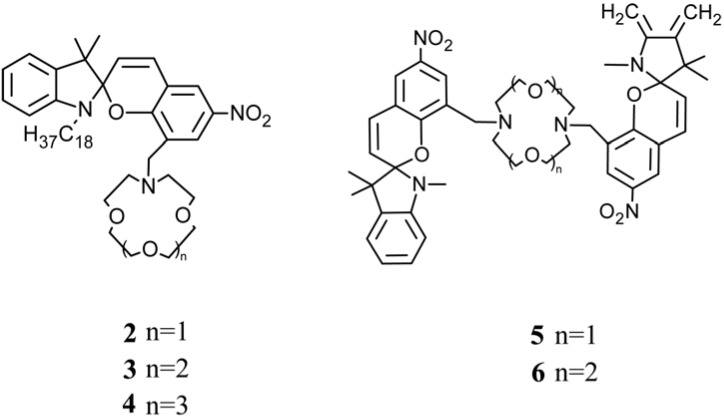
Chemical formula of photo-chromic crown ethers.

Crown ethers present selective cation-complexing properties, especially for alkali and alkaline-earth metal ions [124,125]. The cation-complexing selectivity and sensitivity of crown ethers bearing a photo-chromic functional group can also be controlled and enhanced photo-chemically [121,126]. Thus, photo-chromic crown ethers, as crowned spirobenzopyrans **2–4** and bis-spirobenzopyrans **5**, **6** (Figure 18), are used in metal ion extraction and membrane transport to photo-chemically controllable determination, separation, and sensing of monovalent alkali metal ions [126,127].

**Figure 19:**
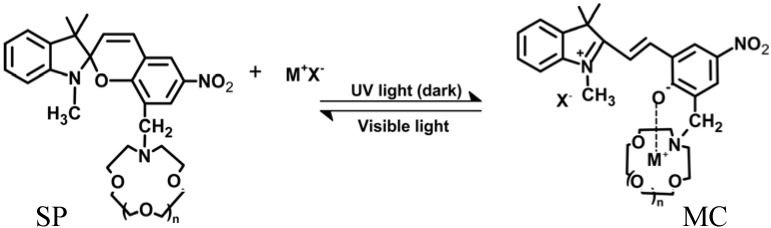
Photo-isomerization of crowned spirobenzopyran.

Crowned spiropyrans **2–4** can be photo-isomerized reversibly to corresponding crowned merocyanine by UV irradiation. Their ability to complex a particular metal ion increases by UV-light and decreases by visible-light irradiation or under dark conditions [127] (Figure 19).

A high charge density on cations promotes specific ionic interactions between MC and metal ions, such as with Li^+^, which contribute to the isomerization because these intramolecular interactions are probably stabilized by six-member chelate formation of crown-ether-ring-nitrogen and phenolate-oxygen atoms with Li^+^. The cation-complexing ability and selectivity of crowned spiropyrans SP are obtained only if the crown ether cavity size matches the cation diameter. This accounts why Na^+^ and K^+^ hardly induce the isomerization. Upon visible light irradiation, the spiropyran form is retained, thus decreasing the Li^ +^ complexing ability, as confirmed by ^7^Li-NMR spectroscopy [126] and electrospray ionization mass spectrometry [127,128].

Multivalent metal cations, such as Ca^2+^ and La^3+^, preferably are complexed by incorporating more than one spirobenzopyran moiety into a crown ether ring (**5**, **6**). UV induced photo-isomerization of crowned bis(spirobenzopyrans) increases the stability of Ca^2+^ and La^3+^ complexes because of the stronger ionic interactions between two phenolate anions of the merocyanine form and metal ions. On the other hand, visible light can switch the ion selectivity of **5**, **6** from La^3+^ to K^+^. 

### 4.3. Photo-Responsive Molecularly Imprinted Membranes

Molecular imprinting has been demonstrated to be a useful technique for the preparation of molecular recognition materials, which have specific binding sites for target molecules [129,130,131,132,133,134,135].

A typical molecular imprinting process usually involves the formation of a complex between a template molecule and a functional monomer via non-covalent interactions, e.g., metal-ion chelating interactions, hydrophobic interactions, hydrogen bonds and ionic interactions, or via reversible covalent bonds, ketal and acetal, Schiff base, or boronate ester. The monomer is subsequently copolymerized with a crosslinking monomer in the presence of a suitable porogenic solvent. After polymerization, the template is removed from crosslinked polymer network, leading to a molecularly imprinted polymer, MIP, with binding cavities complementary to the shape, size and functionality of the template. For the formation of defined recognition sites within MIPs, the structural integrity of the monomer-template assemblies must be preserved during polymerization to allow the functional groups of the polymer to be fixed in space in a stable arrangement that is complementary to the template. However, a polymer matrix must not only contain the binding sites in a stable form, but also be porous enough for easy access of the template (or other analytes) to these sites. Porosity is achieved by carrying out the polymerization in presence of a solvent (a porogen) [136,137,138,139].

Molecularly imprinted polymers have often been compared to antibodies, receptors, and enzymes, and also applied to various fields ranging from analysis to catalysis. Despite the tremendous progress made in the molecular imprinting field, many challenges still remain to be addressed, especially in the design of advanced MIP materials mimicking the biological receptors [140,141,142].

The rational use of certain special co-functional monomers, e.g., *N*-isopropylacrylamide, acrylic acid, and azobenzene monomers, in molecular imprinting together with the appropriate choice of amounts of crosslinkers allows obtaining responsive MIPs towards different stimuli such as temperature, pH, and light [143].

Photo-responsive polymers are a promising route to switchable MIPs. In particular, incorporation of AZB moieties into polymers can result in materials that are photo-responsive [144,145,146,147,148,149].

Minoura *et al.* [150,151] described the first preparation of azo-containing MIP membranes with photo-regulated template binding properties by using a polymerizable derivate of AZB, p-phenylazoacrylanilide as the functional monomer, dansylamide as template and mixtures of tetraethylene glycol diacrylate and ethylene glycol dimethacrylate as cross-linkers. The affinity of dansylamide-specific recognition sites within these MIP membranes can reversibly be changed upon UV or visible light. In this system, binding activity and selectivity were not high, since the functional monomer may not form strong hydrogen bonds with the target molecule.

Marx-Tibbon and Willner, on the other hand, reported the use of another photo-responsive system for molecular-imprinting purposes. They used an acrylate derivatized merocyanine monomer for the imprinting of tryptophan in a molecularly imprinted membrane, which exhibited photo-controllable selective transport properties towards the imprinted amino acid [152].

**Figure 20:**
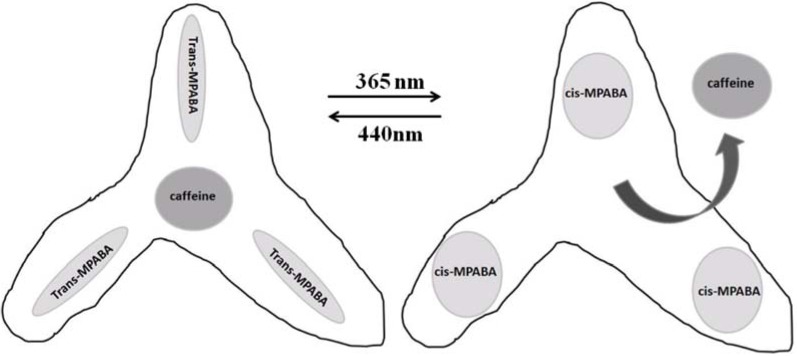
Schematic photo-regulated release and uptake of caffeine.

Gong *et al.* [153] reported the fabrication of a photo-responsive MIP for photo-regulated release and uptake of caffeine by using 4-((4-methacryloyloxy)phenylazo)benzoic acid as the functional monomer. The *trans* → *cis* photo-isomerization properties of 4-((4-methacryloyloxy)phenylazo)benzoic acid are retained after incorporation into the rigid 3D crosslinked polymer matrix. The MIP material enabled to release and take up caffeine molecules upon irradiation at 365 and 440 nm, respectively, as reported in Figure 20. The photo-regulated release of bound caffeine from the MIP material by 365 nm irradiation is attributed to the photo-induced *trans* → *cis* isomerization of AZB chromophores in the MIP receptor sites, resulting in a change in the receptor geometry. The host–guest interaction is therefore weakened, and the bound caffeine is released back into the solution. This can be viewed as an affinity switching of the MIP receptor sites from a high to a low level by photo-isomerization of their azobenzene chromophores. Irradiation of the MIP material at 440 nm after the completion of the photo-regulated caffeine release caused the uptake of caffeine back into the MIP material.

The photo-regulation process is generally reversible, but the extent of substrate release and uptake seems to decrease gradually. This may be caused by the slow deformation of the MIP receptors during the course of repetitive photo-switchings. The results of Gong’s work also suggest the potential of stimuli-responsive MIP materials as smart chemicals and drug-delivery systems.

Later, Gong and coworkers [144,145] prepared photo-responsive molecularly imprinted hydrogels for the photo-regulated release and uptake of pharmaceuticals in aqueous media by using a water-soluble azo-functional monomer, 4-((4-methacryloyloxy)phenylazo)benzenesulfonic acid. *N*-(4-hydroxyphenyl)acetamide, a common analgesic and antipyretic drug known as paracetamol was selected as the molecular template for the imprinting. In this work the authors showed that reorientation of the stimuli-responsive units within the imprinted receptor sites in the hydrogel matrix is sensitive to the nature of the cross-linker used.

Cross-linkers with their polymerizable end-groups separated by longer spacers produce hydrogels that are less restrictive to the stimuli-induced conformation changes of the responsive elements within the materials. In this case, the 4-((4-methacryloyloxy)phenylazo)benzenesulfonic acid-containing polyacrylamide hydrogel fabricated from the cross-linker *N*,*N*′-hexylenebismethacrylamide was found to afford good optical transparency in the aqueous media, reasonable substrate binding affinity, and fast photo-respond rate. Upon irradiation at 353 nm, 83.6% of receptor bound paracetamol was released from the imprinted hydrogel. Subsequent irradiation at 440 nm caused 94.1% of the released paracetamol to be rebound by the hydrogel again. Such a photo-regulated release and uptake process are reversible. 

**Figure 21:**
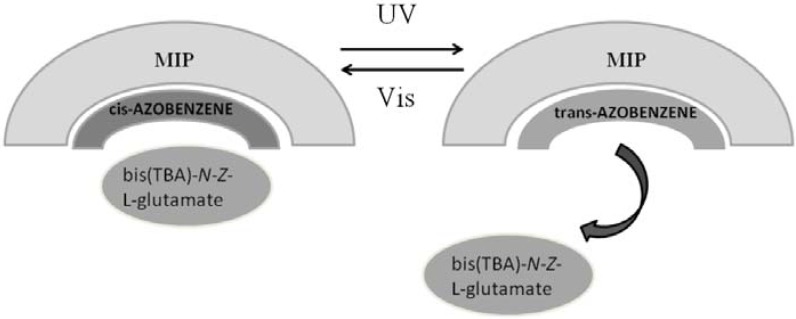
Schematic photo-regulated release and uptake of bis(TBA)-*N*-*Z*-L-glutamate.

Gomy and coworkers [146] developed a new azo monomer di-(ureidoethylenemethacrylate) azobenzene and successfully prepared a photo-responsive MIP. They used bis(TBA)-*N*-*Z*-L-glutamate, a methotrexate analogue, as template. The photo-isomerization properties of the AZB chromophore are retained in the rigid polymer. Photo-induced *cis* → *trans* isomerization of the azobenzene-containing polymer backbone is able to regulate the receptor sites geometry and can regulate the release and uptake of a substrate (Figure 21). This new cross-linking monomer combines interactive monomer functionality with a cross-linking format: it is readily copolymerizable under mild conditions and gives non-covalent MIPs with potential improved performance.

Takeuchi and coworkers [148] synthesized a photo-responsive porphyrin-imprinted polymer by using a novel azo functional monomer bearing a diaminopyridine group. In particular, they used the photo-responsive functional monomer 4-{4-[2,6-bis(*n*-butylamino)pyridine-4-yl]-phenylazo}*-*phenyl methacrylate, FM, for preparing photo-responsive MIP for porphyrin derivatives with carboxylic acids. Multiple hydrogen bonds could be formed between the template and FM, facilitating the assembly of FM with the template in appropriate positions by polymerization and yielding selective imprinted cavities, complementary to the target molecule. The reversible binding of target molecules to the imprinted polymer was directly investigated by using optical waveguide spectroscopy [154,155,156,157,158,159,160].

**Figure 22:**
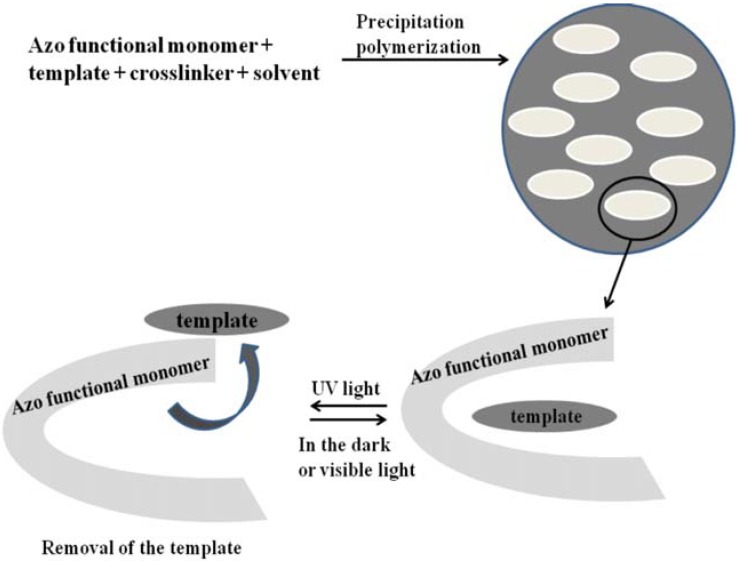
Representation of preparation of azo-containing MIP microspheres with photo-responsive binding sites.

Fang *et al.* [161] described for the first time the successful preparation of azo-containing MIP microspheres with photo-responsive template binding properties. A methacrylate azo functional monomer, with a pyridine group, 4-((4-methacryloyloxy)-phenylazo)pyridine and a good solubility in acetonitrile, allowed the implementation of molecular imprinting via precipitation polymerization, leading to azo-containing MIP microspheres (number-average diameter = 1.33 μm) (Figure 22). 

The binding affinity of the imprinted sites in azo-containing MIP microspheres was found to be photo-responsive towards the template: it decreased upon UV light irradiation, whereas it could be recovered during the subsequent thermal (or visible light-induced) back-isomerization. 

Recently, a novel molecularly imprinted organic–inorganic hybrid AZB material [162] was synthesized by sol–gel route. This MIP was able to release and selectively bind 2,4-dichlorophenoxyacetic acid upon irradiation at 360 and 440 nm, respectively. 

### 4.4. Photo-Responsive Hydrogel Membranes

Environmentally sensitive hydrogels are promising materials for separation and controlled drug delivery. Various stimuli such as pH [163,164,165,166], ionic strength [163,167,168], temperature [169,170,171,172], electric field [167], and magnetic field [173] have been utilized as triggers for drug release.

Andreopoulos *et al.* [174] reported the synthesis of hydrogels via the photo-polymerization of water-soluble poly(ethylene glycol), PEG, molecules. In the later work [175], they examined the diffusive characteristics of light sensitive PEG-based hydrogel membranes by monitoring the permeation rates of model proteins through the membranes. Briefly, the end groups of star-polyethylene glycol molecules were modified with cinnamylidene acetate groups, which crosslink upon exposure to light. Photo-polymerization of cinnamylidene acetate modified PEG molecules, PEG-CA, in water provides a straightforward route to prepare hydrogel membranes in absence of potentially toxic photo-sensitizers and/or photo-initiators. These hydrogels exhibit photo-reversible behavior, and upon exposure to UV light (254 nm) the gels photo-degrade. Consequently, light may be used as a trigger for the release of drugs or other pharmacological agents from a hydrogel matrix. In particular, it has been seen that PEG-CA hydrogels’ swellability is a function of irradiation light (λ > 300 nm) and degree of modification of the PEG molecules. The effect of light on the permeation fluxes of myoglobin, hemoglobin, and lactate dehydrogenase through PEG membranes was also assessed and the diffusion coefficients of the proteins were determined accordingly. Therefore, UV light was used as a trigger to control the mesh size of the membranes, and thereby the permeation fluxes of myoglobin, hemoglobin, and lactate dehydrogenase. Equilibrium swelling experiments with membranes prepared under different irradiation conditions were performed, and the Flory*-*Huggins model was utilized to determine the mesh size and the average molecular weight between crosslinks of the synthesized hydrogels. 

Polyacrylamide hydrogels containing bis-[4-{dimethylamino}phenyl]{4-vinyl-phenyl}methyl leucohydroxide, TPMLH, have been studied as ion selective membranes [176].

In previous works, Irie *et al.* [177,178,179] studied the properties of polyacrylamide, PA, gels containing TPMLH as pendant groups. TPMLH dissociates in a cation (leuco cation) and a hydroxyl anion upon UV light irradiation (270–400 nm). The leuco cation and the hydroxyl anion recombine on sitting in the dark for about 20 h at pH 6.6. 

When PA/TPMLH gels were swollen in pure water, photo-dissociation caused a 3-fold volume increase. This swelling was dependent on the crosslink density and the number of counter ions per bridging polymer chain [180]. Sasaki *et al.* [181] and Willner *et al.* [182] studied the facilitated transport of anionic permeants such as methyl orange by a triphenyl methane derivative in a liquid membrane system. The rate of transport was reported to triple on photo-dissociation of the leucohydroxide. The increased transport was shown to result from the strong binding affinity of the leuco cations for the anionic permeants. This suggests that a photo-responsive ion-exchange membrane could be produced by immobilizing the TPMLH in a polymer matrix. Indeed, Willner *et al.* [183] studied PA/TPMLH gels as enzyme supports and as membranes.

Kodzwa *et al.* [176] studied TPMLH containing polyacrylamide hydrogels as photo-responsive and ion selective membranes. They analyzed factors affecting the ionic diffusion rates based on photo-chromism. They observed that the transport of an anionic permeant was enhanced by the production of photo-cations during irradiation. An increase in methyl orange (anionic) flux after UV irradiation was observed and mainly due to the photo-induced generation of fixed cationic charges in the membrane. On the contrary, the flux of 4-dimethylamino pyridine (a neutral species) was essentially unchanged after irradiation.

### 4.5. Photo-Responsive Polymer Gels

Stimuli responsive polymer gels are some of the most attractive responsive materials because they can be easily tailored to respond to different kinds of stimuli. Non-covalent interactions such as π-π interactions, hydrogen bonds and ionic bonds have been widely explored for preparation of stimuli-responsive polymer gels.

Photo-responsive organogels have been prepared incorporating azo moieties into small molecular gelators [184,185,186,187,188,189,190,191,192] or as pendant groups into main chain polymers [193,194,195,196,197]. Azo groups often exist in tightly-packed aggregates formed by strong intermolecular hydrogen-bonding or π-π interactions. They easily appear in stacking forms named J-aggregates and H-aggregates, [198] which can be distinguished from the spectral shift in their UV-Vis absorption spectra. H-aggregation shows a blue shift due to parallel interaction of azochromophores, whereas a red shift is assigned to J-aggregation (head-to-tail) of azo-groups. H-aggregation has been studied in monolayers, Langmuir-Blodgett films, [199] membranes, bilayers, dispersion, [200] and vesicles [201].

**Figure 23:**
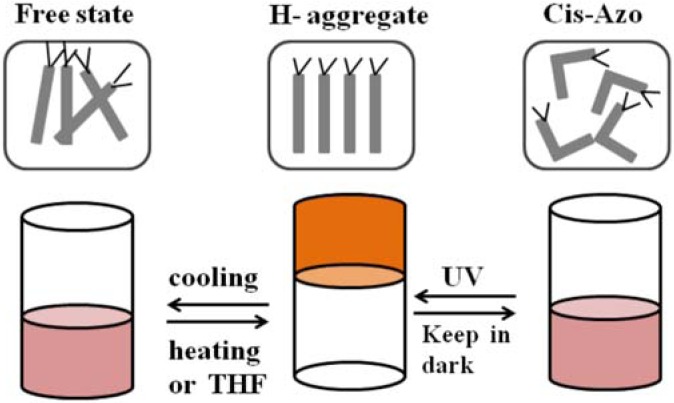
Organogels prepared with PM6AzCOOH in dimethylsulfoxide.

Chen *et al.* [202] prepared multiresponsive reversible gels with a carboxylic azopolymer, PM6AzCOOH. Due to the specifically designed molecular structure of the polymer PM6AzCOOH, the obtained gel showed multiresponsive behaviors to several stimuli, such as temperature, solvent polarity, and light. The specific molecular structure of PM6AzCOOH enables formation of both H-aggregates and hydrogen bonds in solvents with high polarity. The sol-gel phase transition was obtained by heating, or adding solvents with low polarity or UV irradiation (Figure 23). The gel could then be reformed by reverse processes with full reproducibility.

Byrne *et al.* [84] described a polymeric gel modified with a photo-chromic moiety and showed that it is possible to utilize photo-chromic molecules for performing sensing and actuating functions.

Sumaru *et al.* showed that a spiropyran-functionalized poly(*N*-isopropylacrylamide) could undergo photo-sensitive solubility switching [203]. These hydrogels showed the ability to control the permeability of a porous membrane optically [71]. These materials have significant limitations because they cannot operate in an open atmosphere or over a wide range of temperatures, due to water loss from the polymer network. On the contrary, the ability of ionic liquid-based polymer gels to perform such volume phase transitions could usher in a new era of environmentally stable polymer gels, as the swelling/shrinking behavior should be possible in an open atmosphere over much broader temperature ranges and timescales, due to the inherent non-volatility of many ionic liquids. So Byrne *et al.* [84] synthesized photo-responsive ionogels based on poly(*N*-isopropylacrylamide), *N*,*N*-methylene-bis(acrylamide) and protonated benzospiropyran, co-polymerized within several phosphonium based ionic liquids to create hybrid materials for advanced functions. Apart from the advantage of being non-volatile, another advantage of impregnating photo-responsive gels with ionic liquids over aqueous media is the possibility to tailor the properties of the gels using different combinations of anions and cations (polarity, viscosity, *etc.*). Byrne *et al.* observed a photo-responsive contraction of gels under visible irradiation, due to photo-isomerization of the MC−H^+^ to the non-polar BSP isomer that generates a much more hydrophobic environment. As a consequence, bulk water is expelled from the ionogel and causes the physical contraction of the material. These photo-responsive ionogels can be integrated as task-specific optical actuators in microfluidic devices for pumping and valving [204].

### 4.6. Photo-Responsive Dendrimers

Dendrimers are macromolecules made of branched monomers associated around a central core by an iterative process and are characterized by a well defined and highly functionalized 3D architecture. Dendrimers are characterized by a generation number, which refers to the number of repeated branching cycles that are performed during their syntheses. Dendrons are dendritic wedges with one functional group as the core. AZBs can be linked in various places to the structure of dendrimers and dendrons. They can constitute either the terminal groups or the core of the dendrimers (or dendrons), but they can be also incorporated as branches of dendrimers or dendrons. 

AZB group location in dendritic structures affects the isomerization properties: they depend on the physical state of the dendrimers/dendrons (solutions, films, monolayers, nanoparticles, vesicles, fibers, and gels) and the level where the azobenzenes are placed inside the structure (the inner the level, the lower the percentage of isomerization, but the larger the influence on size modifications). The steady state is generally reached after few minutes of UV irradiation and the equilibrium *cis*/*trans* ratio depends on the used wavelength. The *cis* → *trans* tran*s*ition is slower and induced by thermal treatment. The generation number influences the *trans* → *cis* rate, being lower for higher generations. The reverse rate is not sensitive to generation. Also the hydrodynamic volumes are dependent on the isomerization state of AZB groups: the *trans* → *cis* isomerization induces drastic reduction of volume for larger generations [205].

Thin films of AZB dendrimers and dendrons can be obtained by spin coating, and Langmuir-Blodgett techniques. Changes in resistivity, surface area, and molecular hyperpolarizability can be observed upon UV irradiation.

**Figure 24:**
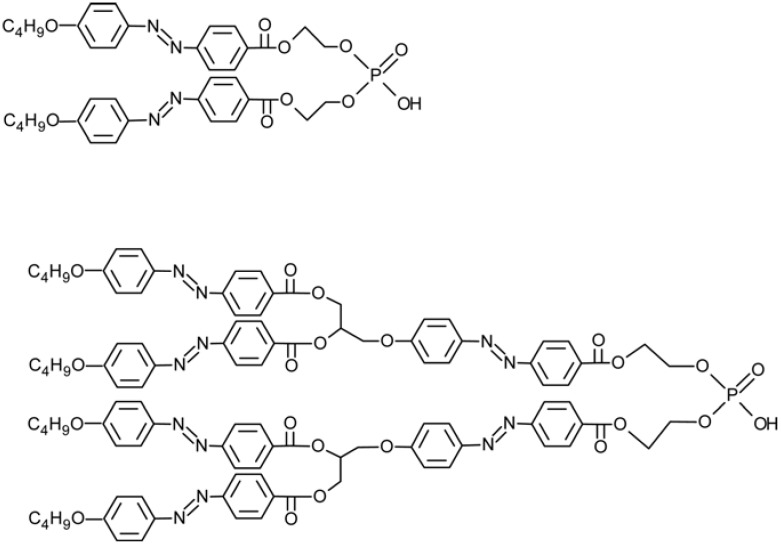
First and second generation of azobenzene-containing dendrimers investigated in [206].

Azobenzene-containing amphiphiles can self-assemble in water into different aggregates such as micelles and bilayer structures, which show interesting photo-responsive properties. The strong interaction of AZB into aggregates is generally confirmed by a spectral shift of the maximum absorption band. A parallel head-to-head alignment (H type) of AZB molecules is detected as a blue shift in the maximum absorption band; on the contrary a red shift is indicative of a head-to-tail arrangement (J type). The different aggregation state strongly affects the aggregate morphology. Dendrimers are polymers with a 3D molecular architecture and a starburst topology, which can bear photo-responsive moieties in order to be sensitive to a light stimulus. As an example, Zhang *et al.* [206] have synthesized from first to the third generation (G0, G1, and G2, see Figure 24) of azobenzene-containing dendrimers with amphiphilic properties and investigated their aggregation behavior in water and light response including morphology changes and drug delivery. 

The aggregates were prepared by both a dilution and sonication method, giving similar dimensions but with a smaller distribution for the latter method. Smaller blue shifts were found for aggregates prepared by sonication indicating that different aggregation state are formed by the two preparation approaches. In fact, the larger blue shifts are indicative for the formation of H aggregates with dilution method, the smaller for looser H-aggregation states. G2 aggregation from the sonication method showed multiple aggregation states. After UV irradiation no significant change was observed in the spectroscopic and morphology behavior of G0 aggregates indicating that the strong H interaction could not be disrupted by UV light. On the contrary, both G1 and G2 aggregate surfaces had significant morphology changes after UV irradiation.

The release of calcein *versus* time was monitored for G1 aggregates upon irradiation. Two different irradiation wavelengths were used: 340 nm responsible for the direct isomerization of H-aggregates and 395 nm responsible for the J-aggregation disruption due to an isomerization of AZB chromophores from *trans* to *cis* state. The release of calcein was faster after a 340 nm irradiation.

Photo-responsive dendrimers and dendrons self-arrange in liquid crystalline structures. In most cases the observed mesophase is the smectic A, while nematic phase is easily found with small dendrimers. Upon UV irradiation, the liquid crystallinity is lost and the systems become isotropic. The irradiation with linearly polarized light induces a photo-orientation of the terminal AZB groups.

Other types of self-assembly are multilayered vesicles, both small and giant ones. Irradiation of AZB dendritic vesicles can induce a photo-controlled release of guest molecules either from the interior or from the membrane of the vesicles, as the *cis* azobenzene moieties in the vesicle membrane enhance the bilayer permeability.

### 4.7. Photo-Responsive Nanoporous Silica Membranes

Periodic mesoporous organosilicas, PMO, including zeolites have been of great interest in the past decade because of their potential applications in membrane separations, adsorption, catalysis, and chemical sensing [207,208,209,210,211,212]. Their ordered periodic architecture defines channels of very regular dimensions in the nanometric scale [213,214,215] with the potential to control pore size and, as a consequence, transport behavior with nanometer-scale precision if appropriate organic functional groups are covalently incorporated onto the pore surfaces of the rigid mesoporous silica framework. PMOs with tunable porosity can serve as size-selective membranes allowing the passage through them of particles and species with dimensions larger than the pores [216,217,218,219,220,221,222,223,224,225]. To expand the range of applications various “switchable” functional groups have been covalently incorporated into PMOs. These membranes with “active” functionality would enable properties to be dynamically controlled by external stimuli, such as light or pH [75,76,215,226,227,228,229,230,231].

Zeolites are transparent 3D-nanostructured porous materials, which are well suited to host guest molecules. AZB molecules have been adsorbed in several zeolite membrane types [229,232] and exhibited reversible photo-switchable permeation properties due to their *trans* → *cis* isomerization. The intrazeolitic photo-isomerization causes changes of molecular size and geometry as well as of dipole moment which affect the permeation of gases such as H_2_, CH_4_, N_2_, O_2_, CO_2_, n-C_4_H_10_, SF_6_, and gas mixtures such as N_2_/CO_2_ and CH_4_/CO_2_. Weh *et al.* [229] measured *trans*/*cis* selectivity, S*_trans/cis_*, and mixture separation factors, α_(__a/b)_, for various gas. S*_trans/cis_* and α_(__a/b)_, were defined as follows:

S*_trans/cis_ =* Permeation (trans)/Permeation (cis)

α_ (a/b)_ = (x_a_/x_b_)_permeate_/(x_a_/x_b_)_retentate_

where x are the mole fractions of gases a and b.

The authors found that results were dependent on the irradiation wavelength, the membrane type, and the amount of adsorbed azobenzene molecules.

Larger permeations of single gas and larger separation factors for equimolar gas mixtures were obtained when AZB molecules were in their *trans* form rather than in their *cis* form. After the switching back to the *trans* form all the permeations increased to the original values. The permeation reversibility was verified over several switching cycles. In addition to steric effects, it was found that electrostatic interactions had to be taken into account for polar gases. S*_trans/cis_* values larger than 2.5 were found for all used gases (except H_2_) in the case of FAU zeolites with larger AZB adsorption as the *cis* form is bulkier and blocks more pore volume. The change in permeation values was more evident for bulky permeant gases (S*_trans/cis_ ≈* 5 for CO_2_). Larger α values were found for N_2_/CO_2_ mixture. Experimental results were in agreement with theoretical prediction of Monte Carlo simulations.

Photo-responsive periodic mesoporous organosilicas can be prepared by means of differently substituted AZB ligands as pendant groups within a porous silica framework. 

**Figure 25:**
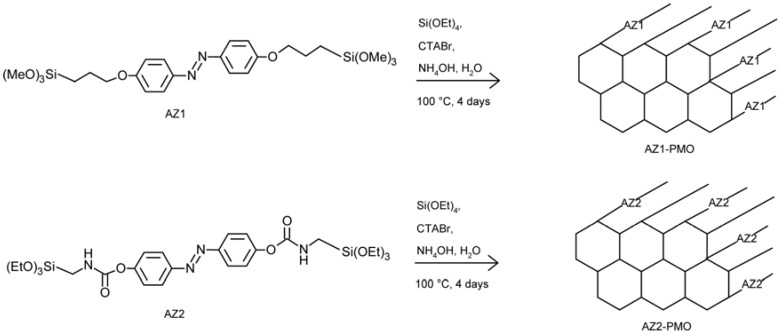
Synthesis paths for AZ1-PMO (**top**) and AZ2-PMO (**bottom**).

PMOs containing photo-active azobenzene moieties covalently linked to the inorganic silica porous structure by propyl linkers, AZ1-PMO, or carbamate groups, AZ2-PMO, were synthesized from tetraethyl orthosilicate (Figure 25). Preparation was carried out under typical synthetic conditions for PMOs using cetyltrimethylammonium as the structure directing agent [233]. Both PMOs showed periodic mesoporous structure as confirmed by powder XRD and isothermal nitrogen adsorptions.

The AZB containing PMOs exhibit a photo-chemical *trans* to *cis* one-way isomerization of the azo group upon irradiation at 358 nm.

The photo-chemical-thermal cycle is reversible and no fatigue of the *trans*/*cis* interconversion has been observed after 5 cycles. As a consequence of *trans*/*cis* isomerization, a reduction of pore size occurs immediately after irradiating for a long time. Variation of the porosity and pore size distribution caused changes in the adsorption capacity of the azobenzene-containing PMOs before and after irradiation. 

Colloidal gold nanoparticles have been also included inside the pores of AZ1-PMO (2.6 nm) and AZ2-PMO (3.0 nm). After irradiation, the gold nanoparticles adsorption capacity of the *cis*-configured azobenzene PMO was significantly increased. The photo-chemical *trans*/*cis* isomerization is thermally reversible and upon storage in the dark the population of *cis*-azobenzene decay back to the *trans* isomer and the adsorption capacity is gradually restored within few days.

Mesoporous materials can be made by surfactant-directed self-assembly [207,208,212].

**Figure 26:**
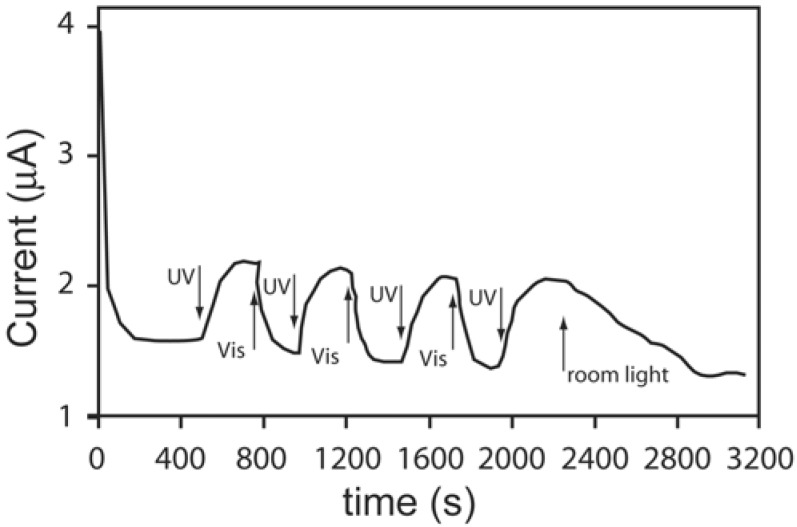
Photo-responsive behavior of nanocomposite film [78] under alternate exposure to UV and visible light.

Liu *et al.* used an azobenzene-modified silane, 4-(3-triethoxysilylpropylureido)azobenzene, to modify the working electrode of an electrochemical cell [78]. The highly ordered mesostructure obtained showed a reversible photo-regulated mass transport to alternate UV and room light exposures. Upon UV irradiation the AZB moieties isomerize to the more compact *cis* form, which increase the pore size and, then, the diffusion rate of probing molecules. Figure 26 shows typical current-time photo-responses. 

## 5. Photoresponsive Membrane Highlights in Technologically Sophisticated Applications

### 5.1. Light-Valved Microfluidics

**Figure 27:**
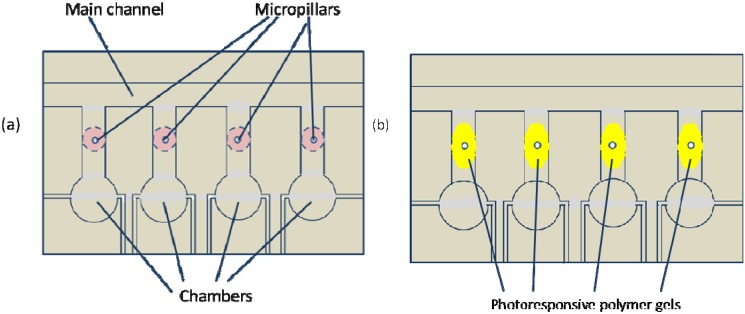
Light-controlled valves.

Amongst key sophisticated technologies, the microfluidics are challenging for integrated analytical systems on microchip. Drug dispensing, transport of liquids, gases and their mixtures, microreactor feeds, microseparation and purification, and movement of liquids at sub-microliter volumes are any few of the attractive examples of molecular machines based on microfluidics. The realization of membrane microvalve is one of the critical issues for the build up of these integrated devices. Light-controlled valves represent an attractive item because flow control by light irradiation enables non-contact fluid control and flow control by local light irradiation enables independent control of multiple fluids. Low-cost biomimetic platforms can be realized using soft polymer actuators in place of external pumps and valves [84]. A poly(*N*-isopropylacrylamide) containing a spirobenzopyran chromophore has been, for example, implanted in a micropattern by *in situ* photo-polymerization at the desired positions in microchannels (Figure 27).

Local light was irradiated onto discrete microvalves allowing independent control of three photo-responsive polymer gel microvalves in a single microchip. This enabled one to open the microvalves within 18–30 s of light irradiation [234]. 

Ionic liquid polymer gels (ionogels) incorporating benzospiropyran units and phosphonium-based ionic liquids have been introduced as photo-actuated valves into micro-fluidic manifolds, forming four micro-valves [204]. The ionogels have been photo-polymerized *in situ* in the channels of a poly(methyl methacrylate) micro-fluidic device and actuated by light irradiation. Depending on ionogel composition each single microvalve can be open at a different time under similar irradiation conditions. A relatively fast opening (few seconds) of the channels and a slow expansion (minutes) suggested this system is practical for single-actuation events. 

### 5.2. Photo-Catalytic Membranes

Organic pollutants found in biologically treated wastewaters have to be removed completely due to public health concerns. Advanced oxidation processes are characterized by the generation of the highly reactive hydroxyl radicals that can tackle and mineralize dissolved organic pollutants.

Among the advanced treatment technologies, TiO_2_/UV photo-catalysis has attracted intensive studies in recent years due to its non-toxic properties [235]. In a photo-oxidation process, hydroxyl radicals are generated when the catalyst, TiO_2_, is illuminated by ultraviolet light and, then, organic compounds are mineralized into CO_2_, H_2_O and inorganic constituents [236,237]. Titanium dioxide is the most commonly used photo-catalyst material due to its strong redox ability, chemical stability, and availability at low cost [238]. Recently, the idea of integrating photo-catalysis with low-pressure submerged membrane has attracted growing attention. Semiconductor photo-catalysis helps to reduce membrane fouling from photo-mineralizing compounds [236,239].

Ho *et al.* [240] investigated the effect of photo-catalytic oxidation coupled with a low-pressure microfiltration in removing effluent organic matter from treated sewage effluents. Integrating photo-oxidation for organic degradation and membrane filtration the authors obtained an enhancement in the filtration flux of the submerged membrane reactor. 

### 5.3. Photo-Responsive PDLCs and Membranes

Among liquid crystal-based systems polymer-dispersed liquid crystals, PDLCs, deserve a particular mention. They are formed by either micron-sized liquid crystal droplets embedded in a polymer matrix (“Swiss cheese” morphology), or by liquid crystal that fills the voids and crevices of a polymer network (“polymer ball” morphology), which gives them a solid-like nature [241]. The physical operation principle of a PDLC is the electrically driven reorientation of liquid crystal directors. In the opaque OFF state of a PDLC, liquid crystal directors are randomy oriented, but they can align parallel to an external electric field and give rise to a transparent device. In both morphologies, the polymer matrix can be considered as the most important component, which gives the device a solid-like characteristic, some important features such as rheological, electrical, and optical properties and the opportunity to host additives to enhance the performances of the obtained devices or to add new functionalities.

Glowacki *et al.* [242] have filled track-etched membranes with cylindrical pores with liquid crystal mixtures doped with mesogenic azo-dyes (14–15 wt%). Liquid crystalline materials act as photo-active elements with response times of a few seconds to UV and visible radiation. In the case of samples with 400-nm pores, membranes have ensured a linear and reversible nitrogen permeability/pressure relationship both in the liquid crystalline and isotropic phase. The nitrogen permeability photo-control was achieved by irradiating samples at 365 and 420 nm. The composition of mesogenic materials and the kind of LC alignment (parallel or perpedicular to pore walls) were shown to influence the nitrogen permeability coefficients through membranes.

**Figure 28:**
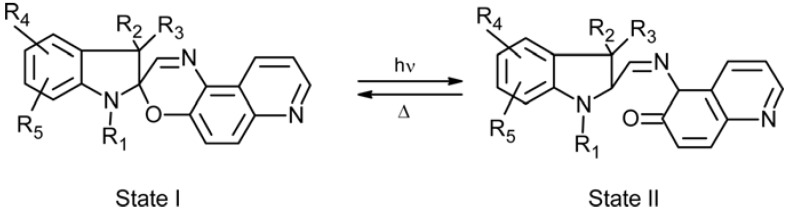
UV-induced structural changes in Photosol^®^ dyes.

Recently, bifunctional films have been obtained. They are able to modulate both the light intensity, by means of the reorientation of the liquid crystal molecules resulting from the application of an external electric field, and the color by UV irradiation, by using small amounts of Photosol^®^ photo-chromic dyes [243,244]. Photosol^®^ dyes are available in various colors and belong to naphthopyran and oxazine classes. Their colorless form contains a spiro-carbon atom which is sp^3^ hybridized and separates the molecules into two halves. In such a geometry (closed form) the molecules absorb in the UV region of the light spectrum (Figure 28 state I). The groups, R_i_, may be selected as described elsewhere [244]. The exposure to ultraviolet radiation causes the rupture of the carbon–oxygen single bond of the oxazine ring (Figure 28 state II). 

As a consequence, the molecular conformation changes into a planar configuration (open form) and the molecular absorption falls in the visible part of the light spectrum. If the UV radiation is removed the carbon–oxygen bond reforms and the molecules relax to the colorless closed form within a few seconds. 

It was possible to tune both the transparency and the color of devices by selecting the UV intensity (color tuning) and the electric field strength (transparency tuning) according the following Table 2. The colored state is achieved in a few seconds on exposure to sunlight or UV, while the bleaching of devices requires several minutes. 

Resistivity is another important property of PDLCs that can be changed by doping polymer matrices with conductive additives. In particular, both nematic emulsions and PDLCs have been doped by small amounts of conductive molecules and oligomers [245,246]. It was found that the electrical conductivity in polymer-dispersed liquid crystals can be finely adjusted and the electric field across the nematic liquid crystal droplets can be increased with a consequent large reduction in the reorientation fields and relaxation times. Furthermore, such simple doping of the polymer matrix does not alter the other properties such as optical and rheological properties of the devices.

**Table 2:** Transparency and color tunability in PDLCs.

	Transparency tuning →			
**↓ Color tuning**	Electric fieldUV intensity	0 V μm^−1^	→	3.5 V μm^−1^
	0%	Transparent and uncolored	→	Opaque and uncolored
	↓	↓		↓
	100%	Transparent and colored	→	Opaque and colored

**Figure 29:**
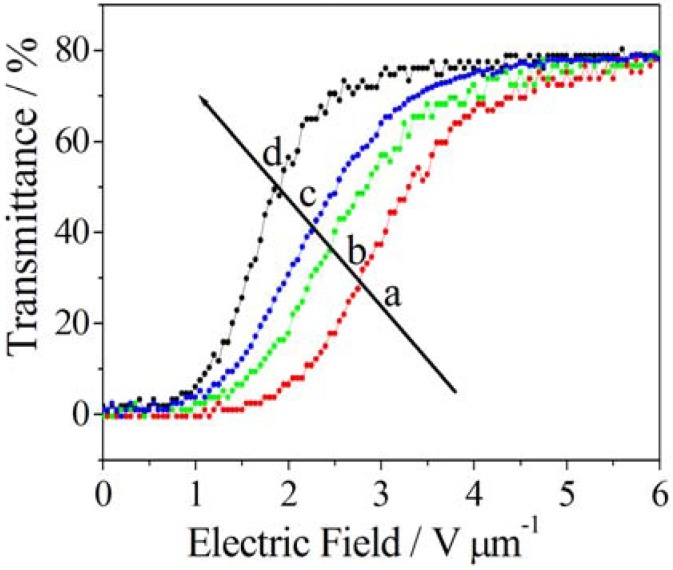
Electric field dependent transmittance as a function of applied UV power: (**a**) 0 mW/cm^2^, (**b**) 2.5 mW/cm^2^, (**c**) 5 mW/cm^2^, and (**d**) 10 mW/cm^2^.

PDLCs can self adjust their transparency by a change of the light intensity [247,248]. Such devices have been fabricated by doping both direct-mode and reverse-mode operation polymer-dispersed liquid crystal films with small amounts of either photo-chromic or photo-conductive molecules (e.g., Photosol^®^ dyes, zinc or dilithium phtalocyanine) in order to change the device conductivity by light in a controlled way and to gain an optically controllable transparency. It was found that in both operation mode films, the electro-optical response changed towards lower switching fields for increasing UV intensity exposures (Figure 29) due to the increase of polymer matrix conductivity. In fact, the field across liquid crystal droplet increased according to the following equation:

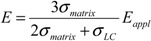
(4)
where *E*_appl_ is the applied external field and σ_matrix_ and σ_LC_ are, respectively, the electric conductivities of polymer matrix and liquid crystal.

**Figure 30:**
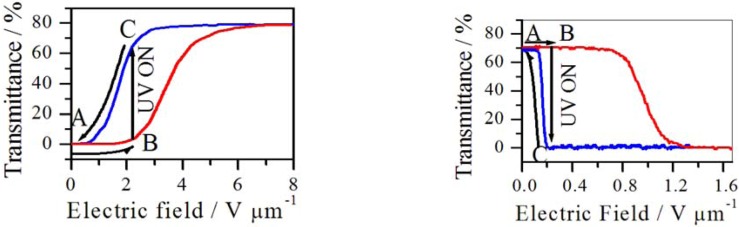
Transmittance dependence on the applied electric field in the dark (red curves) and under UV irradiation (blue curves) for a direct mode PDLC (**left**) and a reverse mode PDLC (**right**).

Figure 30 shows the transmittance dependence on the applied electric field in the dark (curve a) and under UV irradiation (curve b) conditions for a direct-mode (on the left) and a reverse-mode (on the right) operation PDLC. In the dark the initial state A will be opaque/transparent depending on the operation mode of the device. If one applies an electric field lower than the threshold one, in the dark conditions the film will still appear opaque/transparent, but the initial state, A, has moved to an intermediate state, B. After irradiation with a light beam, the film becomes transparent/opaque due to the increase of polymer matrix conductivity and the rise of the effective local electric field across droplets. The device comes back to the opaque/transparent state if either the light source (path C → B) or the electric field (path C → A) is turned off. 

### 5.4. Photo-Electrochromic Systems

It is known that some materials can change color on absorption of light (photo-chromic materials) and others on application of an external electric field (electro-chromic materials). Photo-electrochromics are materials that are able to change their color both on absorption of light and on application of an external electric field.

**Scheme 1:**
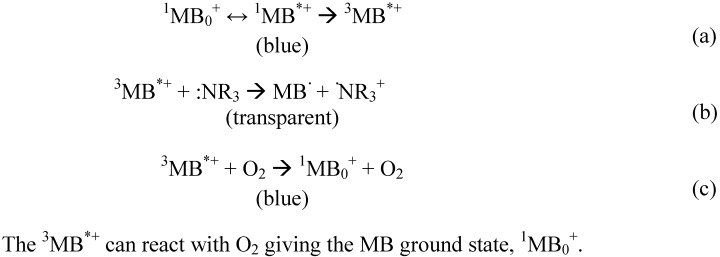
Photo-chromic reactions in methylene blue.

Recently, a new organic photo-electrochromic film has been developed [249,250] in which the same molecule, methylene blue (MB), can change its color from blue to transparent on absorption of Vis light and change back blue thanks to an oxidation reaction induced by an external electric field. The major advantage of such a device is the use of two distinct techniques for performing bleaching and coloring at own will. When a MB molecule absorbs red light (maximum absorption peak at around 660 nm) it undergoes a transition to an excited singlet state, ^1^MB^*+^, which can come back to the initial state, ^1^MB_0_^+^, or convert to an excited triplet state, ^3^MB^*+^. The excited triplet state reduces in the presence of an electron donor species, :NR_3_, changing its initial blue color into a transparent one according to Scheme 1.

At the same time, methylene blue mixed with an oxidative agent in an appropriate solvent can change its color in response to an electrical stimulus. Consequently, it is possible to write on the film by means of a red laser beam and to erase by means of an electrical impulse.

**Figure 31:**
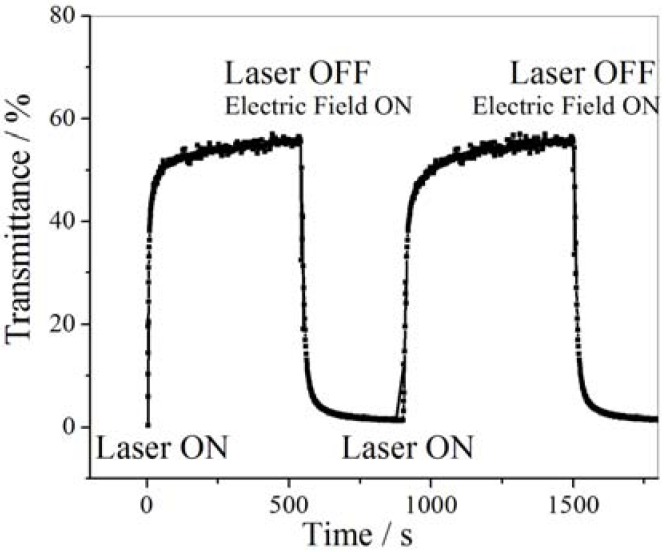
Transmittance behavior on a cyclic red light and electric pulse.

**Figure 32:**
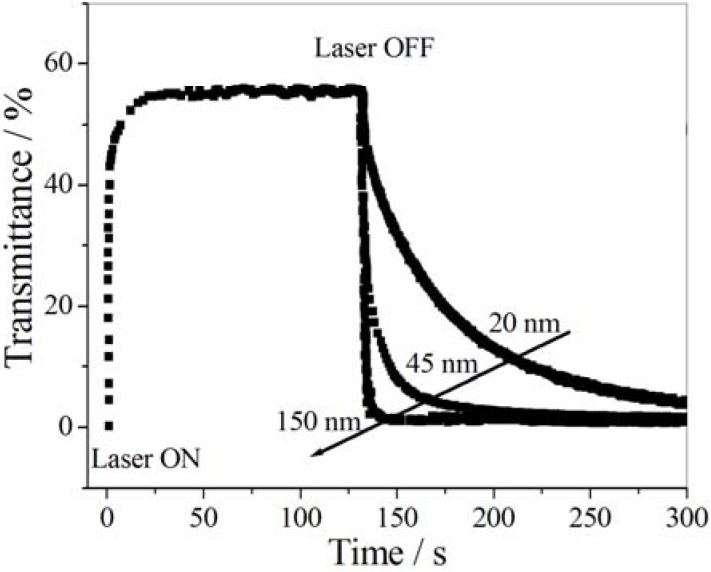
Coloring time as a function of substrate roughness.

When cycling the writing and erasing processes up to 500 times no change in the chromatic properties of films was found (Figure 31). In addition, it was found that:

(1) Different bleaching rates are due to the different electron donor abilities of the amines and the dimensions of the alkyl substituents on the nitrogen. Samples with a tertiary amine show a faster bleaching rate than samples with either a secondary or primary amine. Samples with triethylamine show a faster bleaching rate than samples with tripropylamine or tributylamine.

(2) The coloring time can be reduced by using rougher substrates as they offer a larger surface for the electrochromic reactions (Figure 32).

An important development of the above-described film is reported [251]. In that paper a nanophoto-electrochromic film cast on flexible supports and characterized by faster coloring times was presented.

A colloid solution of TiO_2_ was spin coated on the conductive surface of the PET film and hydrothermally treated. Then phosphate groups were bounded to the surface of the TiO_2_ nanospheres and, finally, the MB molecules to give the PET-TiO_2_-PO_4_-MB film. 

**Figure 33:**
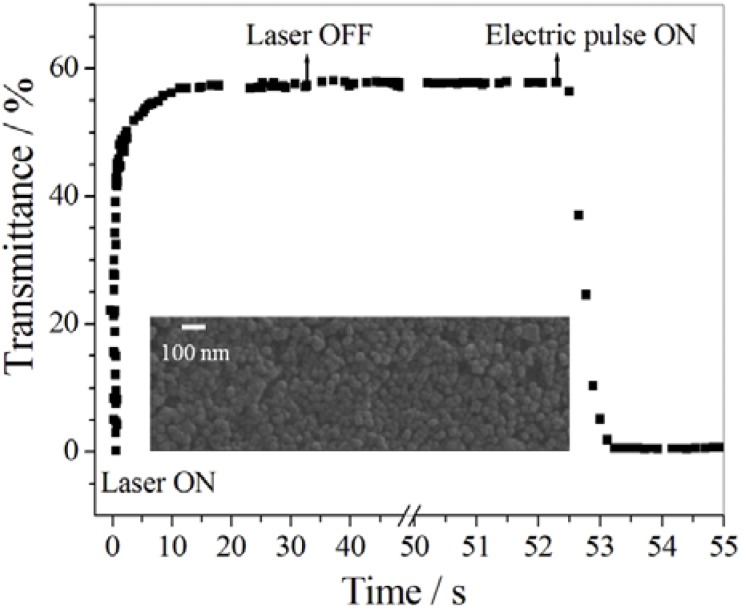
Enhancement in the response of photo-electrochromic devices by using nanostructured electrodes.

The electrolytic solution was absorbed through the pores of a microporous polar membrane. Afterward, the membrane was put between PET-TiO_2_-PO_4_-MB and PET substrates, and the entire device was irradiated with UV light for 5 min to perform the polymerization of the acrylate monomer and to link in a stable way the flexible supports of PET-TiO_2_-PO_4_-MB and PET to the Nafion membrane. In addition to flexibility of PET substrates, the overall process is very fast. Indeed, the oxidation rate is not dependent on the diffusion rate of the molecules toward the electrode. Besides to be able to transport electrical charges, TiO_2_ nanoparticles, because of their dimensions (25 nm in diameter), offer a very high surface area (50 m^2^/g). In this way it has been possible to oxidize a great amount of MB molecules per surface and time unit, Figure 33.

**Figure 34:**
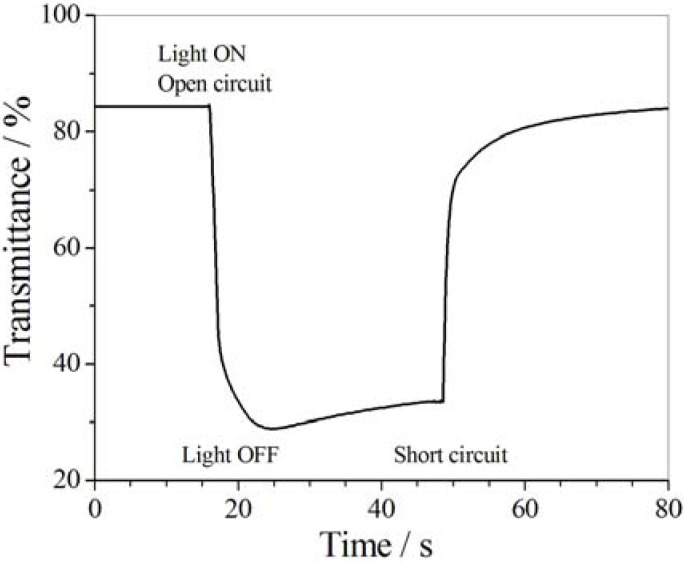
Response of an all-solid photo-electrochromic device.

It is known that the use of conductive polymers allows the manufacture of flexible photo-electrochromic films at low costs [252]. In [253] a new nanostructured and self-supplied photo-electrochromic device was manufactured and characterized. The photo-electrochromic film was obtained by coating dye functionalized TiO_2_ nanoparticles on a layer of WO_3_ nanoparticles acting as an electrochromic layer. In order to improve their electrical conductance, both the dye-TiO_2_ and WO_3_ layers were properly doped with single wall carbon nanotubes bearing COOH groups and a layer of poly(3,4-ethylenedioxythiophene)/poly(styrenesulfonate) was cast between the dye-TiO_2_ layer and the counter electrode. In such a way, all layers were characterized by an increased electrical conductivity, and both coloration and bleaching times were decreased to a few tens of seconds. In addition, an excellent contrast ratio was gained (see Figure 34), and the film had an all-solid nature as no fluid component was used.

## 6. Biological Applications

**Figure 35:**
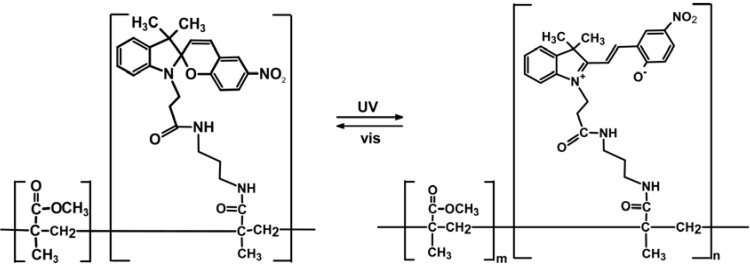
Photo-switching in poly(NSP-co-MMA).

**Figure 36:**
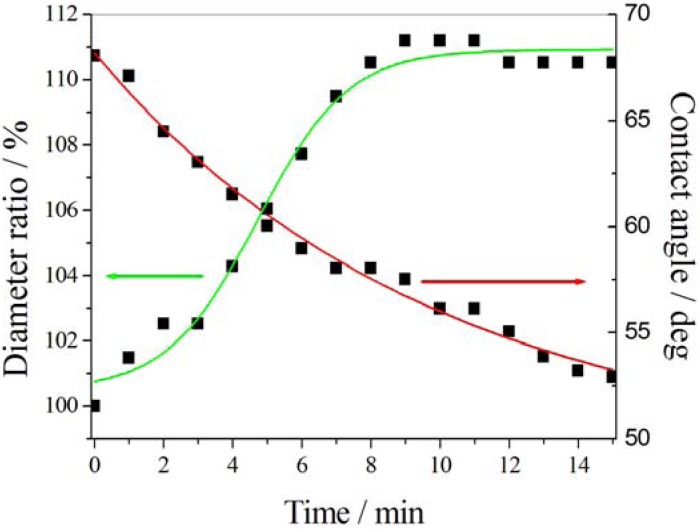
UV induced changes in the hydrophobicity on poly(NSP-co-MMA) coated glass plates.

Photoresponsive membranes find also advanced application in the area of biology. Higuchi *et al.* [254] have synthesized a copolymer of nitrobenzospiropyran and methyl methacrylate, poly(NSP-co-MMA), Figure 35, and investigated the wettability changes of such copolymer films upon irradiation. They observed a significant decrease of water contact angles (≈ −15°) and an increase in the diameter of water droplets (≈ +10%), *i.e.*, an increase of film hydrophilicity, Figure 36. No change was detected in the control surfaces coated with PMMA. Then, they cast glass plates with a poly(NSP-co-MMA) film for platelets and mesenchymal stem cells. The authors found that UV light induced a considerable detachment of both platelets and mesenchymal cells due to the different surface energy between poly(*trans-*NSP-co-MMA) and poly(*cis-*NSP-co-MMA) films and/or to the change in the switching movement of the closed spiropyran form to the open merocyanine form. No significant detachment was found with control pure PMMA films. Similar results were obtained with fibrinogen and related to the switching movement during photo-isomerization.

Another class of attractive soft materials is represented by spherical light-driven membrane gates. These are used, for instance, in many advanced therapies using hydrophobic, poorly water-soluble agents and can seriously be affected by shortcomings such as poor adsorption and bioavailability, embolization of blood vessels, and toxicity. Recently, the photo-isomerization of stilbene chromophore in a photo-responsive dialkoxycyanostilbene polymethacrylate and poly(ethylene oxide) has been demonstrated to be a viable route for the build up of controllable nanodevices for this specific applications [255].

The degradation of self-assembled superstructures was ascribed to the conformational change of stilbene chromophores by UV irradiation. 

**Figure 37:**
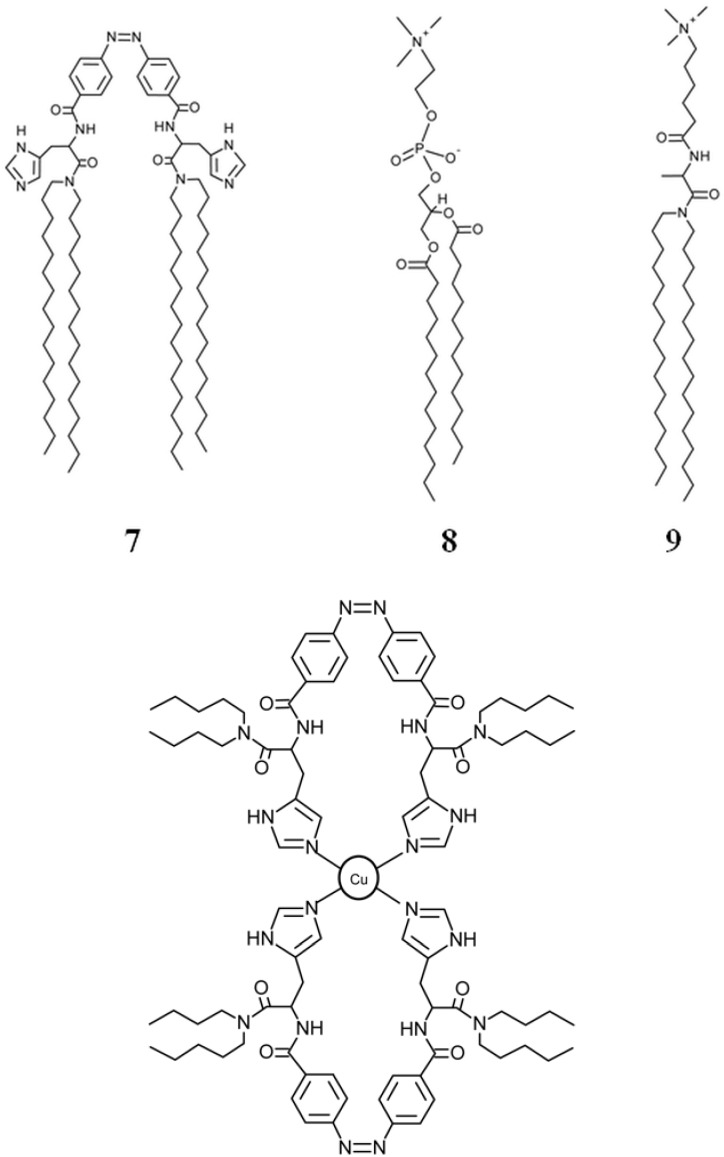
Structural formulas of lipids used in [256] and metal chelates induced by UV irradiation.

**Figure 38:**
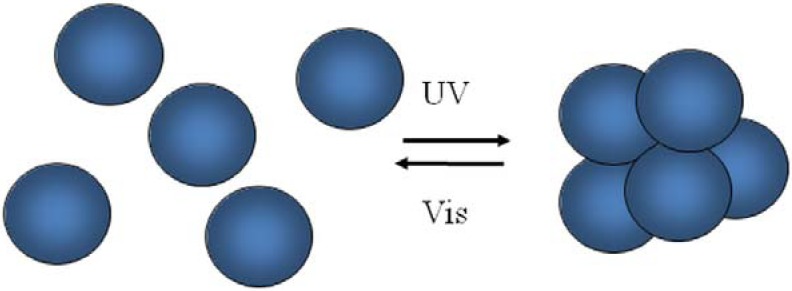
Photo-assembly of liposomes induced by metal chelates from gemini peptide lipids **7**.

Jun-ichi Kikuchi and co-workers [256,257,258] have investigated several systems as molecular switch: as an example, the photo-induced switching behavior of assembly of liposomal membranes with a gemini peptide lipid having L-histidyl residues and an AZB spacer, *N*^2^,*N*^2^’-azobenzene-4,4’-dyol-bis(*N*,*N*-dihexadecyl-L-histidinamide), Figure 37. The authors have used the synthesized gemini peptide lipid as a molecular switch in membranes of phosphatidylcholine liposomes in a solution of Cu^2+^ ions. When the gemini peptide lipids are in their *trans* form (dark conditions) the Cu^2+^-binding affinity of imidazolyl groups is rather low. Upon irradiation with UV light the metal-binding affinity increases due to the photo-isomerization to the *cis*-form and induces the assembly of liposomes, Figure 38. The disassembly of liposomal membranes can be obtained by Vis irradiation and was repeatable in the liquid crystalline state of lipids. The two imidazolyl groups present in each gemini peptide are far more than 0.6 nm apart from each other and, consequently cannot act as a bidentate ligand for a metal ion in the *trans*-form. After UV irradiation (15 min) the *cis*-form is capable of forming metal chelates and inducing assembly among liposomes. This result is confirmed by zeta potential and hydrodynamic diameter (210 nm → 500 nm) changes from vesicles containing the L-histidyl groups.

**Figure 39:**
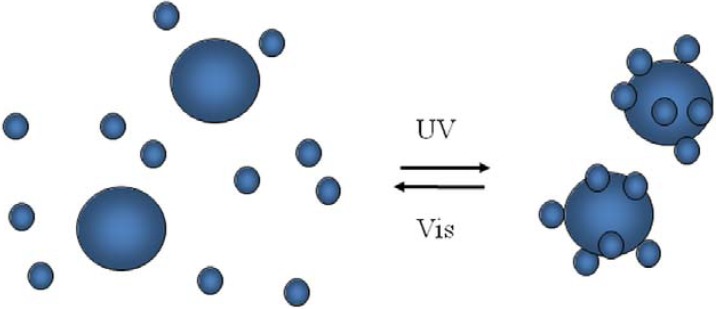
Photo-induced migration and assembly of small liposomes.

On the basis of these results, the authors have built an example of a molecular communication system formed by the previously described liposomes (small and giant dimensions, 100–200 nm and 10 μm, respectively) in an aqueous solution of Cu^2+^ or Zn^2+^ ions. The small liposomes act as a carry information system, while the giant liposomes are the receiver system. Upon UV irradiation (activation of *cis*-form in gemini peptide lipids) the migration and assembly of small liposomes around the giant ones was observed due to the increase of Zn^2+^-binding affinity (Figure 39). The migration was followed by changes in molar circular dichroism, hydrodynamic diameter, and optical microscopy observations. 

**Figure 40:**
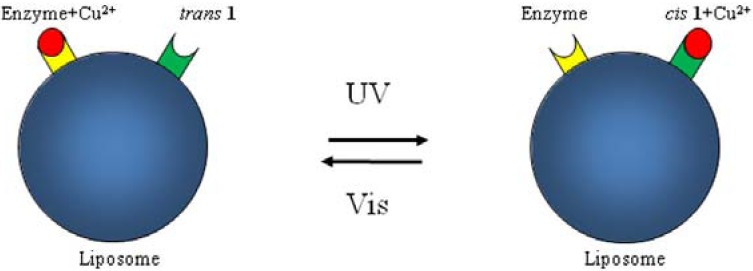
Photo-control of enzymatic activity.

The molecular switch in the receiver was also able to tune the activity of an enzyme. The authors used bilayer vesicles from cationic peptide lipids, *N*,*N*-dihexadecyl-*N*^α^-[6-(trimethylammonium) hexanoyl]-L-alaninamide bromide **(9)**, and gemini peptide lipids in Cu^2+^ aqueous solution. Since cationic bilayer vesicles are able to immobilize L-lactate dehydrogenase [259] and Cu^2+^ ions [260] able to inhibit the enzyme activity, the authors immobilized L-lactate dehydrogenase enzyme on bilayer vesicles and used UV/Vis light to control the binding/release of Cu^2+^ ions and, consequently to amplify/reduce the enzyme activity as shown in Figure 40. Even if the molecular switch has analogous binding affinity towards Cu^2+^ and Zn^2+^ ions, the enzymatic activity was specifically inhibited only by Cu^2+^ ions. 

**Figure 41:**
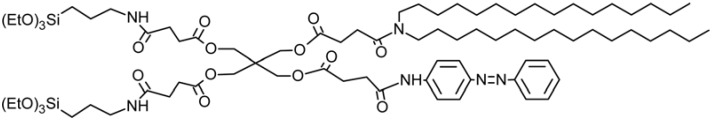
Azobenzene contained organoalkoxysilylated lipid.

Another photo-responsive device was fabricated from an AZB contained organoalkoxysilylated lipid with two triethoxysilyl heads, a hydrophobic double-chain segment, an AZB moiety and a connector unit (Figure 41). Such lipids can self-assemble into photo-responsive cerasomes (spherical vesicles with a 100 nm diameter) with a silicate surface and are able to undergo structural transformation upon UV irradiation, allowing the release of a guest molecule from the liposome membrane. The loading/release properties of liposomes are due to the internal liposomal bilayer structure, which is characterized by low rigidity and density, to the external atomic layer of polyorganosiloxane, which imparts higher morphology stability to cerasomes than that shown by conventional liposomes, and to the photo-isomerizable moieties, which control the release of loaded molecule by light without changing the average liposome size. As a model drug, the release of Nile Red molecules from the hydrophobic compartment to phosphate buffered saline solution was investigated as a function of irradiation time by fluorescence spectroscopy. The UV exposure determines the *cis*-isomerization of photo-responsive tails enhancing the repulsive interactions among amphiphilic moieties and increasing the Nile Red permeability across the membrane. After UV irradiation for 20 min about 50% of Nile Red was released, while no release was observed in dark conditions.

**Figure 42:**
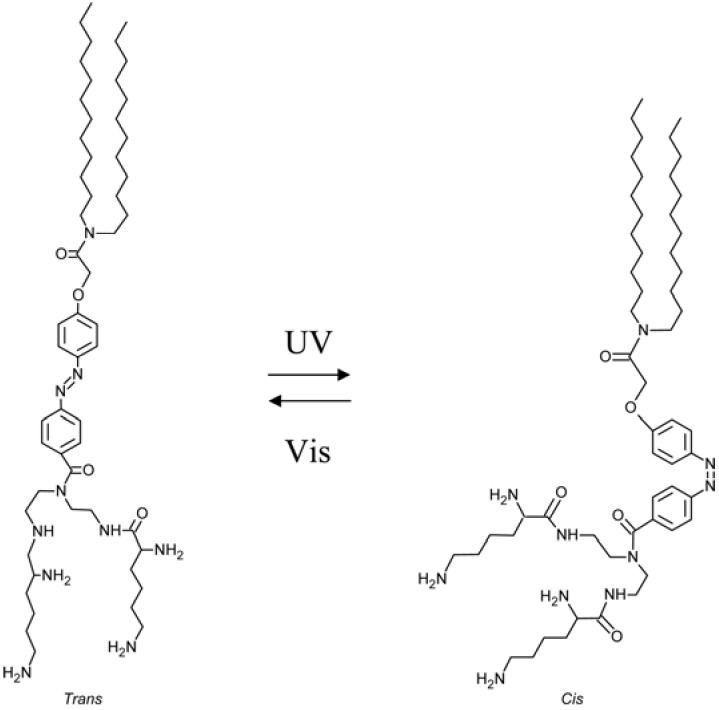
Chemical formula of KAON12.

AZB amphiphiles have been used to control the phase separation in multicomponent giant vesicles [261]. A change in the conformation of the photo-responsive amphiphile (KAON12, 40% mol), according to Figure 42, can switch reversible lateral segregations (a kind of liquid order → liquid disorder transition) in vesicles of lipid mixtures of 1,2-dioleoyl-sn-glycero-3-phosphocholine, 1,2-dipalmitoyl-sn-glycero-3-phosphocholine, and cholesterol (24, 12 and 24 % mol, respectively). Before UV irradiation, *i.e.*, in the *trans* form of KAON12, the membrane surface appears homogeneous without segregation domains; then many small segregation domains appear. The process is reversible by green light irradiation, which “erases” the induced segregations. The phase separation appears as a consequence of a photo-induced increase in the interfacial energy at the domain edges (line tension).

Even in the case of a non-reversible process photo-responsive amphiphiles can be useful in interesting biological applications. Benkoski *et al.* [262] have presented a method for releasing tethered liposomes from a supported lipid bilayer as a function of flow rate and UV irradiation. The tethering is achieved by an amphiphilic molecule with a photo-responsive polymer, poly(2-vinyl-8-hydroxyquinoline-r-8-vinyl-1-naphthoic acid), PVHQ, as the hydrophobic block and DNA as the hydrophilic block. PVHQ shows side groups that can undergo excited state proton transfer in water with a dramatic shift in pKa under UV exposure (90 mW/cm^2^). Upon excitation PVHQ undergoes both the loss of a proton at the hydroxyl group and the gain of a proton on the nitrogen heteroatom in the quinoline ring. The increase in polarity converts the polymer from a lipophilic molecule into a hydrophilic one, changing the partitioning of the molecule in and out of the lipid membrane and allowing the release of tethered liposomes. In their paper the authors tethered 1-palmitoyl-2-oleoyl-sn-glycero-phosphocholinevesicles to a lipid bilayer by a tether of DNA; the ends of which were formed by PVHQ-DNA and cholesterol-DNA conjugates. Liposomes were strongly bound to substrates by a DNA tether in the absence of UV light or in the presence of slow buffer flow. At high flow rate of buffer solution (0.3 mL/s), liposomes desorbed, but in presence of UV light the critical buffer flow to obtain liposome release was reduced to half (0.1–0.2 mL/s). Desorption in the absence of buffer flow may be possible at high UV intensity. No release was observed even at high flow rate if both ends of the tether were formed by cholesterol-DNA conjugates.

**Figure 43:**
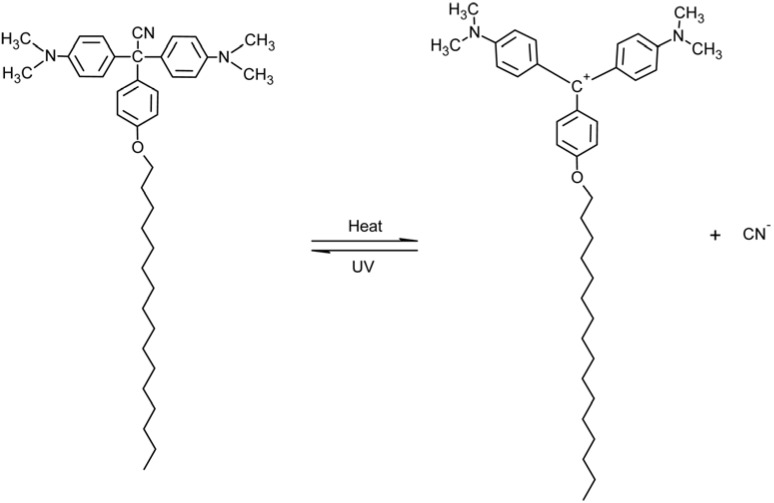
Chemical formula of Malachite Green Leuconitrile derivative.

Photo-responsive amphiphiles can be used to control vesicle formation or disruption in model systems for targeted drug delivery. In such systems, drug molecules contained in vesicles are released when triggered by light as an external stimulus [263,264,265]. A Malachite Green Leuconitrile derivative, MGL, when ionized photo-chemically (Figure 43), exhibits both hydrophilicity and hydrophobicity by its triphenylmethyl cation and its long alkyl chain, respectively. Upon UV irradiation the photo-generated amphiphilicity of MGL can perturb the vesicle bilayer of two single-tailed amphiphiles with oppositely charged head groups consisting of cetyltrimethylammonium chloride, CTAC, and sodium octyl sulfate, SOS [266]. The destabilized bilayer induces a remarkable change in vesicle aggregation depending on the mixing ratio of CTAC/SOS. A small amount of MGL is sufficient to induce an increase of the average size of the vesicle diameter from 116 to 243 nm in the [CTAC]/[SOS] = 0.48 system, whereas this led to an increase in vesicle size polydispersity in [CTAC]/[SOS] = 0.18 system because the bilayer destabilization proceeds through different processes such as disruption, transformation into smaller vesicles and micelles, rearrangement of bilayer packing, and fusion.

Uda *et al.* [267] have characterized the photo-induced morphological changes in phosphatidylcholine vesicles containing small amounts of MGL (less than 5 mol%). Under dark irradiation, the vesicles show spherical with uni- or oligo-lamellar structures. UV irradiation induces non-spherical vesicle morphology. These morphological changes may be accompanied by vesicle fusion (0 < MGL < 1.4 mol%) or photo-induced membrane solubilization (MGL > 2–3 mol%) and the release of vesicle encapsulated drug (or dye) is promoted as the concentration of MGL increases. Membrane solubilization is due to increased destabilization of the vesicle membrane of photo-ionized MGL and is responsible for the opening morphology of the vesicles.

**Figure 44:**
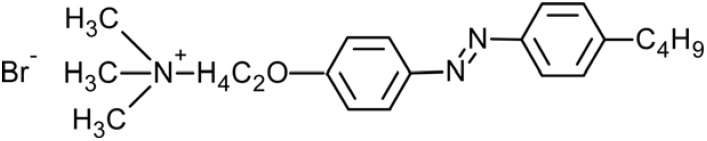
Chemical formula of AZTMA.

Matsumura *et al.* [268] have synthesized a photo-responsive azobenzene-modified amphiphile, 4-butylazobenzene-4’-(oxyethyl)trimethylammoniumbromide (AZTMA), and investigated the photo-chemical control of vesicle disintegration and reformation in aqueous solution by mixing the cationic surfactant AZTMA with an anionic surfactant sodium dodecylbenzenesulfonate, SDBS (Figure 44). 

Vesicles spontaneously form at wide-ranging composition of the *trans*-AZTMA/SDBS system. Upon UV irradiation, the stability of the vesicles is reduced because of increases in the critical packing parameter, CPP, as a result of the bulky structure of *cis*-AZTMA. CPP is given by *V/a*_0_*l_c_*, in which *V* is the volume of the hydrophobic group, *a*_0_ is the surface area of the hydrophilic group, and *l_c_* is the length of the hydrophobic group [269]. The reformation of vesicles was observed after visible light irradiation. In addition, UV light irradiation raised the critical micelle concentrations of AZTMA. Thus, in the AZTMA-rich composition, a solution, which was in a micellar state before light irradiation, changed to a vesicular state after UV light irradiation and visible light irradiation allowed the return to a micellar solution as confirmed by change in the scattered light intensity and the electroconductivity measurements [270].

## 7. Conclusions

This paper reviews the recent progress in light-driven materials and membranes. Different classes of photoswitching and related mechanisms are examined. The advantages of using photo-switching derivates in membranes are discussed. The attractive opportunity to direct changing surface properties towards the construction of molecular machines is examined as well as the construction of programmable valved gates towards selective transport of ions, liquids and gases. Different categories of polymeric adaptive systems are considered and highlighted applications of photo-responsive membranes in biotechnology, chemistry and biology are reported.

## 8. List of Abbreviations

AZB: azobenzene
AZTMA: 4-butylazobenzene-4’-(oxyethyl)trimethylammoniumbromide
BSP: benzospiropyran
CA: contact angle
CD: circular dichroism
CPP: critical packing parameter
CTAC: cetyltrimethylammonium chloride
DMF: *N*,*N*-dimethylformamide
*E*, *E*_appl_: electric field across liquid crystal droplet, applied external field
FM: 4-*{*4-[2,6-bis(*n*-butylamino)pyridine-4-yl]-phenylazo*}-*phenyl methacrylate
*F_y_*: net force on the drop
G0, G1, G2: first, second, third generation dendrimers
h: Planck’s constant
MB: methylene blue
MC: merocyanine
MGL: malachite green leuconitrile
MIP: molecularly imprinted polymer
MMA, PMMA: methylmethacrylate, polymethylmethacrylate
PA: polyacrylamide
PDLC: polymer dispersed liquid crystals
PEG: poly(ethylene glycol)
PEG-CA: cinnamylidene acetate modified PEG
PET: polyethylene terephthalate
PGA: poly(L-glutamic acid)
PMO: periodic mesoporous organosilica
PM6AzCOOH: carboxylic azopolymer
PNA: 2-nitro-4’-methoxyazobenzene
poly(NSP-co-MMA): nitrobenzospiropyran-methyl methacrylate copolymer
PVHQ: poly(2-vinyl-8-hydroxyquinoline-r-8-vinyl-1-naphthoic acid)
SAM: self-assembled monolayer
SP: spiropyran
SPMMA/MMA: spirobenzopyran/methylmethacrylate copolymer
TPMLH: bis-[4-{dimethylamino}phenyl] {4-vinyl-phenyl} methyl leucohydroxide
SDBS: sodium dodecylbenzenesulfonate
SOS: sodium octyl sulfate
S_trans/cis_: trans/cis selectivity
*V*: volume of the hydrophobic group
*w*: drop width
*a*_0_: surface area of the hydrophilic group
*l_c_*: length of the hydrophobic group
UV: ultraviolet light
Vis: visible light
α_(__a/b)_: mixture separation factor
Δ: heat or thermal relaxation
ΔE: energy band-gap
ν, ν’: frequency
θ: contact angle value
*γ_LV_*: surface free energy of the liquid-vapor interface
*γ_SL_*: surface free energy of the solid-liquid interface
*γ_SV_*: surface free energy of the solid-vapor interface
σ_matrix_ , σ_LC_: polymer matrix electric conductivity, liquid crystal electric conductivity
